# Three novel *Rothia* species associated with Antarctic birds harbour novel biosynthetic gene clusters

**DOI:** 10.1093/femsec/fiag056

**Published:** 2026-05-29

**Authors:** Vendula Koublová, Jana Musilova, Karel Sedlar, Peter Spacek, Jitka Vives, Eva Staňková, Ondrej Šedo, Stanislava Kralova, Ludek Sehnal, Ivo Sedláček, Pavel Švec

**Affiliations:** Department of Experimental Biology, Czech Collection of Microorganisms, Faculty of Science, Masaryk University, Kamenice 5, 625 00 Brno, Czech Republic; Czech Academy of Sciences, Global Change Research Institute, Bělidla 986/4a, 603 00 Brno, Czech Republic; Department of Biomedical Engineering, Faculty of Electrical Engineering and Communication, Brno University of Technology, Technická 12, 616 00 Brno, Czech Republic; Department of Biomedical Engineering, Faculty of Electrical Engineering and Communication, Brno University of Technology, Technická 12, 616 00 Brno, Czech Republic; Department of Molecular Pharmacy, Faculty of Pharmacy, Masaryk University, Palackého třída 1946/1, 612 00 Brno, Czech Republic; Department of Chemistry and Biochemistry, Faculty of AgriSciences, Mendel University in Brno, Zemědělská 1665/1, 613 00 Brno, Czech Republic; Department of Experimental Biology, Czech Collection of Microorganisms, Faculty of Science, Masaryk University, Kamenice 5, 625 00 Brno, Czech Republic; Department of Experimental Biology, Czech Collection of Microorganisms, Faculty of Science, Masaryk University, Kamenice 5, 625 00 Brno, Czech Republic; Central European Institute of Technology, Masaryk University, Kamenice 5, 625 00 Brno, Czech Republic; Department of Molecular Pharmacy, Faculty of Pharmacy, Masaryk University, Palackého třída 1946/1, 612 00 Brno, Czech Republic; Department of Chemistry and Biochemistry, Faculty of AgriSciences, Mendel University in Brno, Zemědělská 1665/1, 613 00 Brno, Czech Republic; Department of Experimental Biology, Czech Collection of Microorganisms, Faculty of Science, Masaryk University, Kamenice 5, 625 00 Brno, Czech Republic; Department of Experimental Biology, Czech Collection of Microorganisms, Faculty of Science, Masaryk University, Kamenice 5, 625 00 Brno, Czech Republic; Department of Experimental Biology, Czech Collection of Microorganisms, Faculty of Science, Masaryk University, Kamenice 5, 625 00 Brno, Czech Republic

**Keywords:** *Rothia*, antarctica, penguins, genomic analysis, biosynthetic gene clusters, polyphasic taxonomic approach

## Abstract

Antarctica's unique environment supports diverse avian populations, including penguins, skuas, and gulls. As climate-driven pressures on these populations intensify, their microbiota receives increased attention due to their relevance for host health. In this study, we investigated 11 bacterial isolates associated with Antarctic birds using a polyphasic taxonomic approach integrating genomic and phenotypic data. Phylogenetic analysis of 16S rRNA gene sequences and core-genome-based phylogenomics separated the strains into three groups and genome-relatedness indices confirmed that the three lineages represent novel species within the genus *Rothia*. Functional genomic analysis, combined with phenotypic testing, revealed broad metabolic capabilities with adaptations to host-associated environment. Further genome mining revealed the presence of several biosynthetic gene clusters, potentially encoding terpenes, siderophores, and other bioactive compounds. Some of these clusters likely encode variants of enterobactin, a nonribosomally synthesized siderophore. However, in vitro production of enterobactin was not confirmed, suggesting more complex expression regulation than iron depletion alone. Together, these findings broaden the known diversity of *Rothia* by three proposed novel species, *Rothia ornithocola* sp. nov., *Rothia pygoscelis* sp. nov., and *Rothia antarctica* sp. nov., and highlight genomic and phenotypic features that contribute to the ecology of Antarctic avian-associated bacteria.

## Introduction

Antarctic ecosystems support a diverse array of avian species, including penguins, skuas, and gulls, many of which occupy dense breeding colonies and exhibit complex social and trophic interactions. These birds play key roles in the Antarctic food web and biogeochemical cycles, and their health and population dynamics are increasingly affected by environmental change (Sailley et al. 2013, Goldenberg-Barbosa et al. 2025). In this context, avian-associated microbial communities are gaining attention as integral components of host biology. Most research on the microbiota of Antarctic birds has focused on penguins, reflecting their ecological prominence and accessibility (Grzesiak et al. 2020, Kong et al. 2023, Kaczvinsky et al. 2024, Goldenberg-Barbosa et al. 2025).

Previous studies have provided valuable insights into the taxonomic composition and, to a limited extent, the functional potential of penguin-associated microbial communities (Dewar et al. 2013, Yew et al. 2017, Grzesiak et al. 2020, Zeng et al. 2021, Kaczvinsky et al. 2024). Most of these studies have focused primarily on the gut or faecal microbiota (Dewar et al. 2013, Yew et al. 2017, Wen Chyin et al. 2018, Grzesiak et al. 2020, Zeng et al. 2021, Kaczvinsky et al. 2024, Goldenberg-Barbosa et al. 2025), which are highly relevant for understanding microbial dispersal and the formation of ornithogenic soils in Antarctic environments (Wen Chyin et al. 2018). In contrast, few studies have addressed the oral microbiota of penguins (Saunderson et al. 2021, Graciette et al. 2023, Kong et al. 2023, Švec et al. 2023, Koublová et al. 2024), despite its relevance to individuals' health, feeding behaviour, and chick development (Saunderson et al. 2021). Studies on other Antarctic birds, such as skuas and kelp gulls, remain scarce and are restricted mainly to surveys of antimicrobial resistance genes (Liakopoulos et al. 2016, Ewbank et al. 2021) or the detection of particular microbial taxa in faecal samples or carcasses (Leotta et al. 2006, Johansson et al. 2018, Hubálek 2021).

This study deals with bacteria from the genus *Rothia*, phylogenetically assigned to the family *Micrococcaceae* within the phylum *Actinomycetota*. The genus was established in 1967 to accommodate *Rothia dentocariosa*, a species previously classified within the genera *Nocardia* and *Actinomyces* (Austin 2015). To date, 14 *Rothia* species with validly published names have been described (LPSN, retrieved May 2026) (Parte et al. 2020).

Several *Rothia* species are common inhabitants of the human oral cavity (Uranga et al. 2020, Baker et al. 2021), while others have been associated with infections in humans, particularly in immunocompromised patients (Franconieri et al. 2021, Getzenberg et al. 2021, Bissell et al. 2022). Beyond humans, members of the genus have also been detected in a wide range of animal hosts, including chickens (Gilroy et al. 2021, Zhang et al. 2022), geese (Wang et al. 2021, Kang et al. 2022), woodpeckers (Braun et al. 2018), ostriches (Abolnik et al. 2021), hummingbirds (Herder et al. 2021), mullet fish (Jammal et al. 2017), mice (Collins et al. 2000), pigs (Oliveira et al. 2022), and sloths (Rojas-Gätjens et al. 2022). In addition to animal-associated habitats, *Rothia* species have been isolated from diverse environmental sources, such as plants (Xiong et al. 2013, Shurigin et al. 2020, Tuikhar et al. 2022), fermented foods (Park et al. 2010, Jeong et al. 2016), soil (Chou et al. 2008), wastewater sludge (Fan et al. 2002), and air (Li Y et al. 2004, Kämpfer et al. 2016), highlighting the ecological versatility of the genus.

Members of the genus *Rothia* are known to interact with other microorganisms in their environments and their hosts through a variety of mechanisms, including nitrite production (Mazurel et al. 2023), cross-feeding (Gao et al. 2018), suppressing the inflammatory response (Rigauts et al. 2022), and the production of antimicrobial secondary metabolites (Oliveira et al. 2022, Rojas-Gätjens et al. 2022, Stubbendieck et al. 2023). Genomic analyses have revealed the presence of diverse biosynthetic gene clusters (BGCs) in *Rothia* genomes (Gaiser et al. 2017, Oliveira et al. 2022, Rojas-Gätjens et al. 2022, De La Cruz et al. 2024), and antagonistic activity against bacteria and fungi has been demonstrated *in vitro* (Rojas-Gätjens et al. 2022, De La Cruz et al. 2024). However, the chemical identity and structural diversity of the produced molecules remain poorly characterized. Based on sequence similarity, some of the encoded compounds are thought to be related to valinomycin, a cyclododecadepsipeptide ionophore with antibiotic activity (Gaiser et al. 2017), enterobactin, an iron-chelating siderophore (Uranga et al. 2020, Akomoneh et al. 2023, Stubbendieck et al. 2023), linocin, a peptide with antimicrobial activity (Rojas-Gätjens et al. 2022), beta-lactones, heterocyclic compounds with antimicrobial potential (Rojas-Gätjens et al. 2022, You et al. 2022), and actinorhodin, a pH sensitive antimicrobial pigment (Akomoneh et al. 2023). Among these metabolites, siderophores may play a particularly important ecological role in *Rothia* spp. They are high-affinity iron-chelating compounds that facilitate iron acquisition under iron-limited conditions, including host-associated environments where iron availability is restricted by nutritional immunity. Beyond iron uptake, siderophore production can contribute to microbial competition, colonization, oxidative stress tolerance, and persistence within microbial communities (Harrison and Buckling 2009, Johnstone and Nolan 2015, Kramer et al. 2020). The identification of enterobactin-like pathways across multiple *Rothia* genomes therefore suggests that iron acquisition may represent an important but still understudied aspect of *Rothia* ecology and adaptation.

Several *Rothia* species also exhibit resistance to various antimicrobial agents. Multidrug-resistant strains have been reported to harbour genes conferring resistance to a broad range of antibiotic classes, including beta-lactams, aminoglycosides, macrolides, sulfonamides, fluoroquinolones, rifamycins, tetracyclines, lincosamides, trimethoprim, and chloramphenicol (Wang et al. 2021, Oliveira et al. 2022).

In this study, we present comprehensive phenotypic and genomic analyses of 11 *Rothia* strains isolated from Adélie and Gentoo penguin oral swabs, Antarctic bird faeces, and bird nesting sites. These analyses included phylogenetic and phylogenomic reconstruction, functional genome annotation, and BGC analysis, providing insights into both evolutionary relationships and the potential ecological roles of the studied strains within Antarctic avian-associated ecosystems. Extensive phenotypic characterization, including metabolic capabilities, tolerated growth conditions, and susceptibility to antibiotics, was subsequently performed to support and contextualize the genomic findings. Based on the combined genomic and phenotypic evidence, we propose that these strains represent three novel bacterial species belonging to the genus *Rothia*.

## Materials and methods

### Strains and isolation

A total of 11 bacterial strains (Supplementary Table S1) were included in this study. These strains were isolated from beak swabs of Adélie penguins (*Pygoscelis adeliae*) and Gentoo penguins (*Pygoscelis papua*), as well as from faecal samples of Antarctic birds, including skua (*Stercorarius* sp.) and kelp gull (*Larus dominicanus*). Sampling was conducted on James Ross Island and Marambio Island during multiple expeditions (annually, 2011–2021) to the Johann Gregor Mendel Czech Antarctic Research Station.

Beak swabs were collected from adult penguins using sterile swab kits containing Amies medium with charcoal (COPAN Italia) and stored at 4°C until processed. Swabs were streaked onto Tryptone Soya agar (TSA, Oxoid) plates and incubated aerobically at 30°C for up to 5 days. Colonies exhibiting distinct morphologies were continuously picked and purified through subsequent streak plating. Selected strains were deposited in the Czech Collection of Microorganisms (CCM, Masaryk University, Brno, Czech Republic) and BCCM/LMG bacteria collection (Ghent University, Ghent, Belgium). The strains were grown aerobically on TSA plates at 30°C for 2 days for all the following analyses, unless specified otherwise.

For comparative purposes, three reference strains were selected based on 16S rRNA gene sequence similarity and phylogenomic relationships and obtained from Czech Collection of Microorganisms: *Rothia nasimurium* CCM 9216^T^, *Rothia endophytica* CCM 9215^T^ and *Rothia amarae* CCM 9402^T^.

### Rep-PCR fingerprinting

Repetitive sequence-based PCR (rep-PCR) (Versalovic et al. 1994) using the (GTG)_5_ primer was performed for the initial genotyping of the Antarctic bird-associated strains. Genomic DNA was extracted using an alkaline lysis protocol as described previously (Švec et al. 2008). Rep-PCR amplification, gel electrophoresis, and fingerprint analysis were carried out following established protocols (Švec et al. 2010). Fingerprints were analyzed with BioNumerics 7.6 (Applied Maths), and a dendrogram was calculated using Pearson's correlation coefficient and the UPGMA clustering method.

### 16S rRNA gene sequencing and phylogenetic analysis

The nearly full-length 16S rRNA gene was amplified using primers pA and pH (Edwards et al. 1989) as previously described (Švec et al. 2012) using the same genomic DNA extract as for rep-PCR. PCR products were purified using the High Pure PCR Product Purification Kit (Roche), and Sanger sequencing was performed by Eurofins Genomics (Germany) using the same primer set. As this procedure failed to yield reads of sufficient quality for strains P7162^T^, P7181, and P13129, primer pair 616 V and 1492R was used under cycling conditions previously described (Loy et al. 2002) using genomic DNA extracted with High Pure PCR Template Preparation Kit (Roche). PCR amplicons were purified using ExoSAP-IT™ Express PCR Product Cleanup (Applied Biosciences) according to the manufacturer's instructions. Sanger sequencing was performed using the EZ-Seq service (Macrogen) with the same set of primers. Sequencing reads were assembled with the CAP3 tool (https://doua.prabi.fr/software/cap3/) (Huang and Madan 1999), and the resulting sequences were compared to the EZBioCloud database (https://ezbiocloud.net/) (Yoon et al. 2017). The 16S rRNA gene sequence similarities between the studied strains were calculated using BLAST (https://blast.ncbi.nlm.nih.gov/) (Altschul et al. 1990).

Phylogenetic analysis was conducted using MEGA11 (Tamura et al. 2021) with 18 reference sequences obtained from the NCBI GenBank database. *Micrococcus luteus* DSM 20030^T^ (AJ536198.1) was used as an outgroup. MUSCLE was used for multiple sequence alignment. The evolutionary relationships were inferred using the Maximum Likelihood (Felsenstein 1981) method based on the Tamura-Nei model (Tamura and Nei 1993), with 1000 bootstrap replications (Felsenstein 1985). A discrete Gamma distribution (five categories, +G, parameter = 0.1059) was used to model rate variation among sites, and invariable sites were allowed ([+I], 43.80% sites). Positions with <95% site coverage were excluded (partial deletion option).

Additional phylogenetic trees were reconstructed using Minimum Evolution (Rzhetsky and Nei 1992) and Neighbor-Joining (Saitou and Nei 1987) methods with the Tamura–Nei model (Tamura K and Nei 1993) and 1000 bootstrap replicates (Felsenstein 1985). Rate variation among sites was modelled with a Gamma distribution (shape parameter = 0.52) as determined by the MEGA11 model selection tool. Ambiguous positions were removed for each sequence pair (pairwise deletion option).

### MALDI-TOF MS protein profiling

Protein fingerprinting was conducted using Matrix-Assisted Laser Desorption/Ionization—Time of Flight Mass Spectrometry (MALDI-TOF MS) with an Ultraflextreme instrument (Bruker Daltonics) following ethanol/formic acid extraction (Freiwald and Sauer 2009). A MALDI-TOF mass spectra-based dendrogram was constructed using Pearson's correlation coefficient as a measure of similarity and UPGMA as a grouping method.

### Genome sequencing and assembly

Genomic DNA for whole-genome sequencing was extracted using the High Pure PCR Template Preparation Kit (Roche). Strains P5764, P5766, P6271, and P7182^T^ (= CCM 9418^T^) were sequenced in collaboration with RECETOX (Masaryk University, Brno) and CEITEC Genomics Core Facility (Masaryk University, Brno). DNA was quantified using Qubit 2.0 with the dsDNA High Sensitivity kit (Invitrogen), and libraries were prepared using the Nextera XT DNA Library Preparation kit (Illumina) according to the manufacturer's protocol. Sequencing was performed on an Illumina MiSeq instrument with the MiSeq Reagent Kit v2.

Strains P4278, P5758 (= CCM 9416), P5771^T^ (= CCM 9417^T^), P7208, P7162^T^ (= CCM 9419^T^), P7181, and P13129 were sequenced at CEITEC Genomics Core Facility (Masaryk University, Brno). Input DNA was quantified using Quantus with Quantifluor dsDNA kit (Promega).

Libraries were prepared using 200 ng of total DNA with the xGen DNA EZ Library Prep Kit (IDT) following the manufacturer's protocol, with 16-min fragmentation time and six PCR cycles. AMPure SPRIselect beads (Beckman-Coulter) were used for DNA purification, and indexing was performed using xGen UDI primer sets. Libraries were quantified, pooled, and sequenced on Illumina NovaSeq S6000 in paired-end 2 × 161 bp mode. Libraries failing to yield sufficient reads were converted to MGI sequencing libraries by circularization and sequenced on the MGI G400 platform using paired-end 2 × 150 bp mode.

Raw reads were quality-assessed using a combination of FastQC v0.12.1 and MultiQC v1.7 (Ewels et al. 2016). Adapter sequences and low-quality bases were trimmed using fastp v0.24.0 (Chen 2023). Genomes were assembled using Unicycler v0.5.0 (Wick et al. 2017) and the final report was generated by the QUAST tool (Gurevich et al. 2013). The quality of the resulting assemblies was assessed using CheckM2 version 1.1.0 (Chklovski et al. 2023) and database version 1.0.2 within the Galaxy platform (https://usegalaxy.cz/) (Chudoba et al. 2017).

### Comparative pan-genome analysis

**Figure 1:**
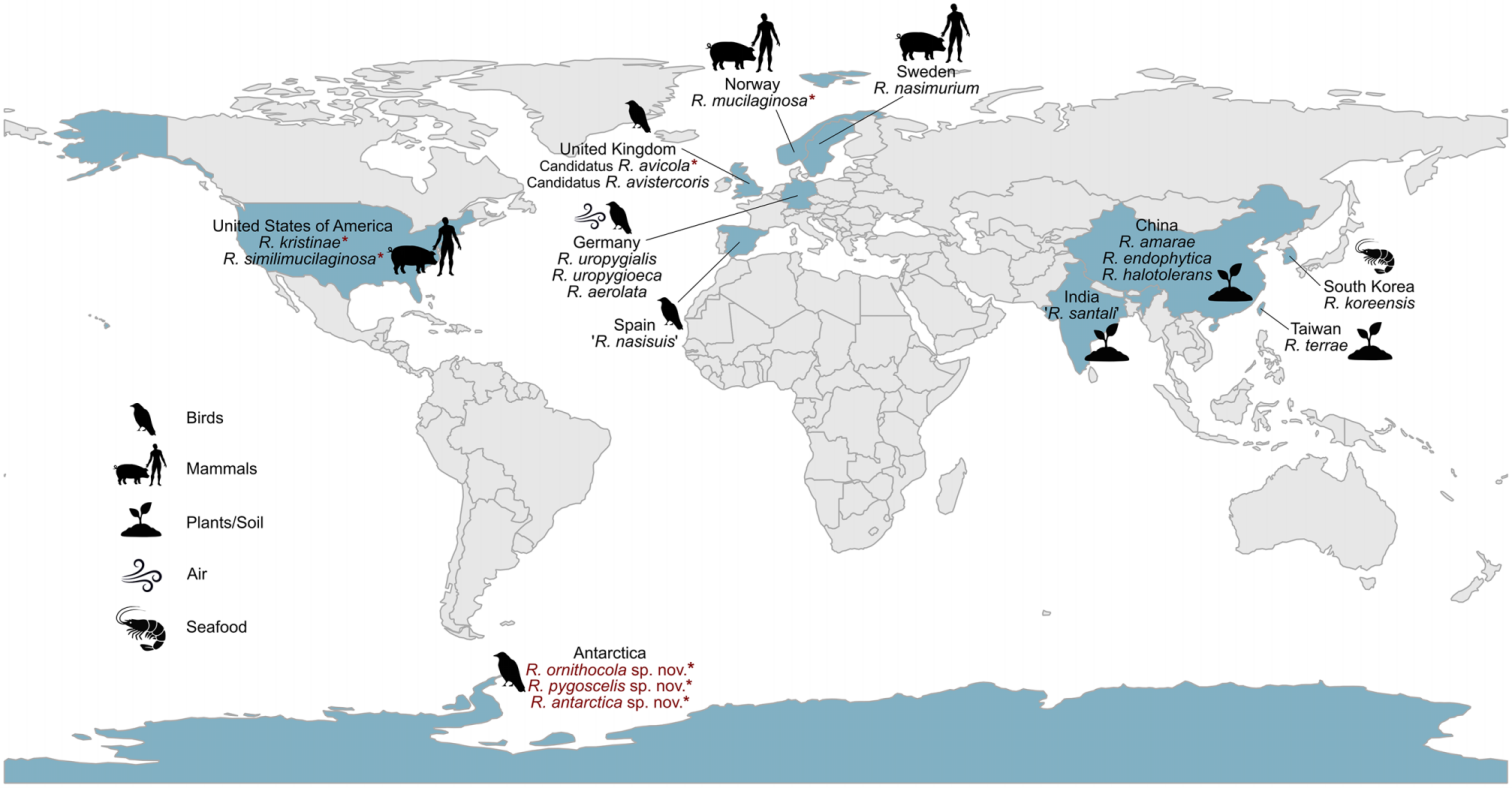
Global distribution and ecological origin of representative type strain genomes used for comparative analysis in this study, alongside the 11 Antarctic isolates. An asterisk indicates the presence of an enterobactin-like biosynthetic gene cluster (BGC) in the corresponding genome. Type strain of *Rothia dentocariosa* was originally isolated from a human caries lesion, with no geographical origin specified in the original description. Type strain of *Rothia aeria* was isolated from air samples collected aboard Mir space station.

Eighteen reference *Rothia* genomes were retrieved from NCBI GenBank (accessed 8 May 2026) (O’Leary et al. 2016), including genomes of the type strains of all validly named *Rothia* spp., two non-validly published species "*Rothia nasisuis"* 69RC1 and "*Rothia santali*" AR01^T^, as well as two metagenome-assembled genomes (MAGs) of *Candidatus* species, "*Candidatus* Rothia avicola" and "*Candidatus* Rothia avistercoris". Accession numbers of all genomes used in comparisons are provided in Supplementary Table S3. Fig. 1 shows geographical origin of the 18 reference genomes and the 11 Antarctic isolates.

Pan-genome analysis was performed with BPGA v1.3.0 (Chaudhari et al. 2016), with amino acid sequences clustered using USEARCH v11.0.667 (Edgar 2010), with an identity cut-off of 50%. The concatenated sequences of core genes were aligned with MUSCLE v3.8.31 (Edgar 2004). A distance matrix was calculated using the Kimura substitution model, and a phylogenomic tree was reconstructed using the Neighbor-joining method (Saitou and Nei 1987) and visualized in MEGA11 (Tamura Koichiro et al. 2021).

### Average nucleotide identity and digital DNA–DNA hybridization

Average nucleotide identity (ANI) was calculated using the OrthoANIu tool (Yoon et al. 2017) and visualized in RStudio (R Core Team 2024, Posit team 2025) using ComplexHeatmap (Gu et al. 2016), circlize (Gu et al. 2014), and viridis (Garnier et al. 2023) packages. Digital DNA–DNA hybridization (dDDH) values were calculated for all Antarctic strains and 18 *Rothia* reference genomes included in the study (Supplementary Table S3) using Genome-to-Genome Distance Calculator v3.0 (https://ggdc.dsmz.de) (Meier-Kolthoff et al. 2013), employing formula 2 as recommended.

### Phenotypic characterization

Gram staining was performed using the Poly Stainer System (IUL Instruments), and cell morphology was examined under a BX53 light microscope (Olympus). Gram staining results were confirmed using the KOH lysis test (Moaledj 1986).

Growth on tryptone soya agar (TSA; Oxoid) and Columbia blood agar with 5% sheep blood (Oxoid) was assessed by spreading 50 µl of inoculum (∼2 McFarland) on the medium and incubating at 30°C for up to 48 h. The temperature range supporting growth was determined in tryptone soya broth (TSB; Oxoid) at 5°C, 10°C, 15°C, 20°C, 25°C, 30°C, 37°C, 40°C, 45°C, and 50°C. Salinity tolerance was tested in TSB enriched with 5%, 6%, 7%, 8%, 9%, 10%, and 11% NaCl (w/v).

The following phenotypic properties were tested: motility (Bennett et al. 1979, Lennette et al. 1980), production of oxidase (OXItest, Erba Lachema), catalase (ID Colour Catalase, bioMérieux), and acetoin (VPtest, Erba Lachema), pyrrolidonyl arylamidase (PYRAtest, Erba-Lachema), β-galactosidase (ONPG test) (Lowe 1962), urease (Christensen 1946) and lecithinase activity (egg yolk reaction) (Owens 1974), nitrate reduction (Barrow and Feltham 1993), and hydrolysis of DNA (CM321, Oxoid), aesculin (Barrow and Feltham 1993), Tween 80, gelatine (Páčová and Kocur 1984), tyrosine (Kurup and Babcock 1979) and starch (Barrow and Feltham 1993). Additional enzymatic and biochemical profiling was performed using STAPHYtest 24 (Erba Lachema) and API ZYM (bioMérieux) according to the manufacturers' instructions. All tests were inoculated either directly from 2 days old cultures aerobically grown on TSA, or a ∼0.5 McFarland solution prepared from the same cultures.

### Susceptibility to antibiotics

Antibiotic susceptibility was determined by the disc diffusion method on Mueller-Hinton agar (Oxoid) according to the EUCAST standards (EUCAST 2024: 14). Representatives of various antibiotic classes were tested: ampicillin (10 µg), cefepime (30 µg), ciprofloxacin (5 µg), clindamycin (2 µg), erythromycin (15 µg), moxifloxacin (10 µg), gentamicin (10 µg), chloramphenicol (30 µg), linezolid (10 µg), imipenem (1 µg), penicillin G (1 IU), rifampicin (5 µg), cotrimoxazole (25 µg), tetracycline (30 µg), and vancomycin (15 µg). As standardized breakpoint criteria for *Rothia* spp. using the disc diffusion method are not available (CLSI 2020, EUCAST 2024), interpretive criteria were adopted from EUCAST guidelines, version 14.0 (EUCAST 2024) for phylogenetically related genera, including *Corynebacterium* (ciprofloxacin, clindamycin, erythromycin, moxifloxacin, linezolid, penicillin G, rifampicin, cotrimoxazole, tetracycline, and vancomycin), *Staphylococcus* (ampicillin, gentamicin), *Streptococcus* (cefepime, chloramphenicol), and *Enterococcus* (imipenem). This approach is consistent with the CLSI M45 guidance for *Rothia mucilaginosa*, where interpretive criteria (except for penicillin) are derived from *Staphylococcus* spp. Specific breakpoint values used are provided in Supplementary Table S5.

### Fatty acid methyl esters analysis

Fatty acid methyl esters (FAME) extraction and analysis were performed using an Agilent 7890B gas chromatograph according to the Sherlock MIDI Identification System protocol (MIDI Sherlock version 6.2; MIDI database, RTSBA 6.21).

Bacterial cultures were grown on TSA (BD Difco) at 30±2°C until late exponential phase: 24 h for P4278, P5758 (= CCM 9416), P5764, and P5771^T^ (= CCM 9417^T^); 48 h for P5766 and reference strains *R. endophytica* CCM 9215^T^, *R. nasimurium* CCM 9216^T^, and *R. amarae* CCM 9402^T^; and 72 h for P6271, P7182^T^ (= CCM 9418^T^), P7208, P7162^T^ (= CCM 9419^T^), P7181, and P13129, following the four-quadrant streak method (Sasser 1990).

### Functional genomic analysis

Genome annotation included both structural and functional aspects. Initially, structural annotations were carried out using the NCBI Prokaryotic Genome Annotation Pipeline (PGAP) (Tatusova et al. 2016).

Functional annotation included KEGG Orthology and Links Annotation (KOALA) performed using BlastKOALA v3.1 (Kanehisa et al. 2016) and assignment of Clusters of Orthologous Groups (COGs) categories using eggNOG-mapper v2.1.12 with the eggNOG database v5.0 (Cantalapiedra et al. 2021). Prophage sequences were searched using online version of PHASTER (Arndt et al. 2016), and clustered regularly interspaced short palindromic repeat (CRISPR) loci were detected in the annotated genome sequences using CRISPRDetect v2.4 (Biswas et al. 2016). In addition, restriction-modification (R-M) systems were detected using DefenseFinder (Tesson et al. 2024) in protein sequences. The resistome was predicted using nucleotide fasta sequences with the Resistance Gene Identifier (RGI) 6.0.3 against the CARD 4.0.0 database (Alcock et al. 2023), allowing loose hits and a ≥95% identity nudge.

### Biosynthetic gene cluster analysis

Biosynthetic gene clusters (BGCs) encoding secondary metabolites were identified using the stand-alone version of antiSMASH v8.0.0 (antibiotics and Secondary Metabolite Analysis Shell), with the parameters *–genefinding-tool prodigal* and *–hmmdetection-strictness relaxed* (Blin et al. 2025). For comparative analysis, 18 reference *Rothia* spp. genomes and MAGs were analyzed (Supplementary Table S3). To investigate the evolutionary relationships among the enterobactin BGCs within the identified gene cluster family (GCF 3), a phylogenetic analysis was performed using BiG-SCAPE derived alignments based on conserved protein domains shared within each GCF (Draisma et al. 2026). Maximum-likelihood phylogenetic trees were reconstructed using IQ-TREE v3.1.1 (Wong et al. 2026). The best-fit substitution model was selected using ModelFinder according to the Bayesian Information Criterion (BIC) (WAG+F+R3), and branch support was assessed using 1000 ultrafast bootstrap replicates.

### Siderophore assays

To detect and quantify iron-chelating activity during growth, siderophore production was evaluated using the universal Chrome Azurol S (CAS) assay in a 96-well microplate format (Schwyn and Neilands 1987, Arora and Verma 2017). Cultivation conditions were adapted from a previous study demonstrating enterobactin production in *R. mucilaginosa* ATCC 25296^T^ under iron-limited suboptimal M9 medium conditions supplemented with glucose or saccharose (Uranga et al. 2020). Strains were cultivated in 6 ml of M9 medium, and culture supernatants were collected after 3, 5, and 7 days of incubation. Supernatants were subsequently analyzed using both the CAS assay and Arnow's assay to confirm the production of catecholate-type compounds with 100 µM catecholate as a standard (Arnow 1937). To additionally assess siderophore production during surface-associated growth, CAS-M9 agar plates were prepared, inoculated, and incubated at 30°C for 7 days prior to evaluation of colour-change zones indicative of iron chelation (Louden et al. 2011). *Rothia mucilaginosa* CCM 2417^T^ was used as a positive control. All experiments were performed in three independent biological replicates.

### Environmental dispersal

Environmental dispersal of the studied species was assessed using the Branchwater web interface (https://branchwater.jgi.doe.gov, 27 April 2026) (Irber et al. 2022). Whole-genome FASTA assemblies of all 11 strains were uploaded as separate queries against the indexed NCBI Sequence Read Archive (SRA) metagenome collection, using default search parameters with the minimum containment threshold lowered to 0.05. Match quality was evaluated using the reported k-mer-based containment (the fraction of query k-mers detected in the metagenome) and the derived containment-based average nucleotide identity (cANI), interpreted against the conventional 95% ANI threshold for species delineation (Chun et al. 2018) and the recommended Branchwater reliability cut-off of containment 0.1.

## Results and discussion

### Rep-PCR fingerprinting

As part of the initial screening of a larger strain collection, rep-PCR fingerprinting was applied for strain grouping and dereplication of clonally related isolates. All studied strains, as well as the reference strains, yielded distinct rep-PCR profiles, confirming that the isolates represent unique, non-clonal strains. Cluster analysis of the fingerprints revealed that the Antarctic bird-associated isolates formed three coherent groups, clearly separated from each other as well as from other entries in the in-house database and from the reference *Rothia* strains included in the analysis (Supplementary Fig. S1).

### 16S rRNA gene sequencing and phylogenetic analysis

Partial 16S rRNA sequences (1347–1401 bp) were obtained for all studied strains and deposited in the NCBI database under accession numbers PV222019–PV222029 (Supplementary Table S1). The comparison of the obtained sequences against the EZBioCloud database yielded results suggesting that the 11 Antarctic strains belong to genus *Rothia* (Table 1). Most of the obtained values were below the generally accepted threshold for species delineation of 98.7% (Chun et al. 2018), indicating that the studied strains represent novel species within the genus *Rothia*.

**Table 1:** Pairwise 16S rRNA gene sequence similarities (%) between the eleven Antarctic *Rothia* isolates and the six most closely related *Rothia* type strains.

Strain	*Rothia nasimurium* CCUG 35957^T^	*"Rothia marina"* JSM 078 15^1^	*Rothia endophytica* YIM 67072^T^	*"Rothia nasisuis"* 1a5R-CH16^T^	*Rothia terrae* L-143^T^	*Rothia amarae* JCM 11375^T^
**P4278**	98.92	98.05	97.98	97.98	97.69	97.60
**P5758 (= CCM 9416)**	99.19	98.39	98.32	98.24	97.73	97.57
**P5764**	98.70	97.84	97.77	97.91	97.70	97.47
**P5766**	98.92	98.06	97.99	97.99	97.70	97.48
**P5771^T^ (= CCM 9417^T^)**	98.64	97.78	97.71	97.71	97.64	97.47
**P6271**	96.68	97.26	97.19	96.97	96.76	97.24
**P7208**	96.98	97.55	97.48	97.27	96.84	97.33
**P7182^T^ (= CCM 9418^T^)**	96.75	97.32	97.25	97.04	96.68	96.88
**P7162^T^ (= CCM 9419^T^)**	97.18	97.47	97.40	97.32	96.89	97.69
**P7181**	96.87	97.16	97.09	97.02	96.58	97.38
**P13129**	97.10	97.40	97.33	97.25	97.40	98.14

Similarity values were calculated using the EZBioCloud 16S-based identification service (Yoon et al. 2017). Strains are grouped by proposed novel species: *R. ornithocola* sp. nov. (P4278, P5758 = CCM 9416, P5764, P5766, P5771^T^ = CCM 9417^T^), *R. pygoscelis* sp. nov. (P6271, P7182^T^ = CCM 9418^T^, P7208), and *R. antarctica* sp. nov. (P7162^T^ = CCM 9419^T^, P7181, P13129).

Strains P4278, P5758, P5764, P5766, and P5771^T^ shared 99.85%–100% sequence similarity, strains P6271, P7182^T^, and P7208 shared 99.86–99.93%, and strains P7162^T^, P7181, and P13129 shared 99.63%–100%. Sequence similarities between the first group and second and third group were considerably lower, ranging from 96.80%–97.01% and 96.88%–97.40%, respectively. In contrast, the second and third groups shared notably high mutual similarities of 99.41%–99.85%.

Based on 16S rRNA gene sequence similarity, three *Rothia* type strains were selected as references for phenotypic testing. *R. nasimurium* CCUG 35957^T^ was included due to its high similarity to the first group of strains (P4278, P5758, P5764, P5766, and P5771^T^). For the remaining two groups, *R. amarae* JCM 11375^T^ and *R. endophytica* YIM 67072^T^ were selected, as one of the most closely related species, “*Rothia marina*”, has not been validly published (LPSN, retrieved December 2025; Parte et al. 2020).

The isolation of *Rothia* strains from Antarctic birds was not unexpected, as different *Rothia* species have previously been reported from a broad range of avian hosts (Braun et al. 2018, Abolnik et al. 2021, Herder et al. 2021, Kang et al. 2022, Zhang et al. 2022). However, to our knowledge, *Rothia* has not yet been documented in the oral microbiomes of Adélie or Gentoo penguins, which remain poorly studied (Kong et al. 2023). Moreover, we were unable to identify any studies addressing the faecal microbiomes of kelp gulls or skuas, highlighting a substantial gap in current knowledge of microbial communities associated with Antarctic birds.

**Figure 2:**
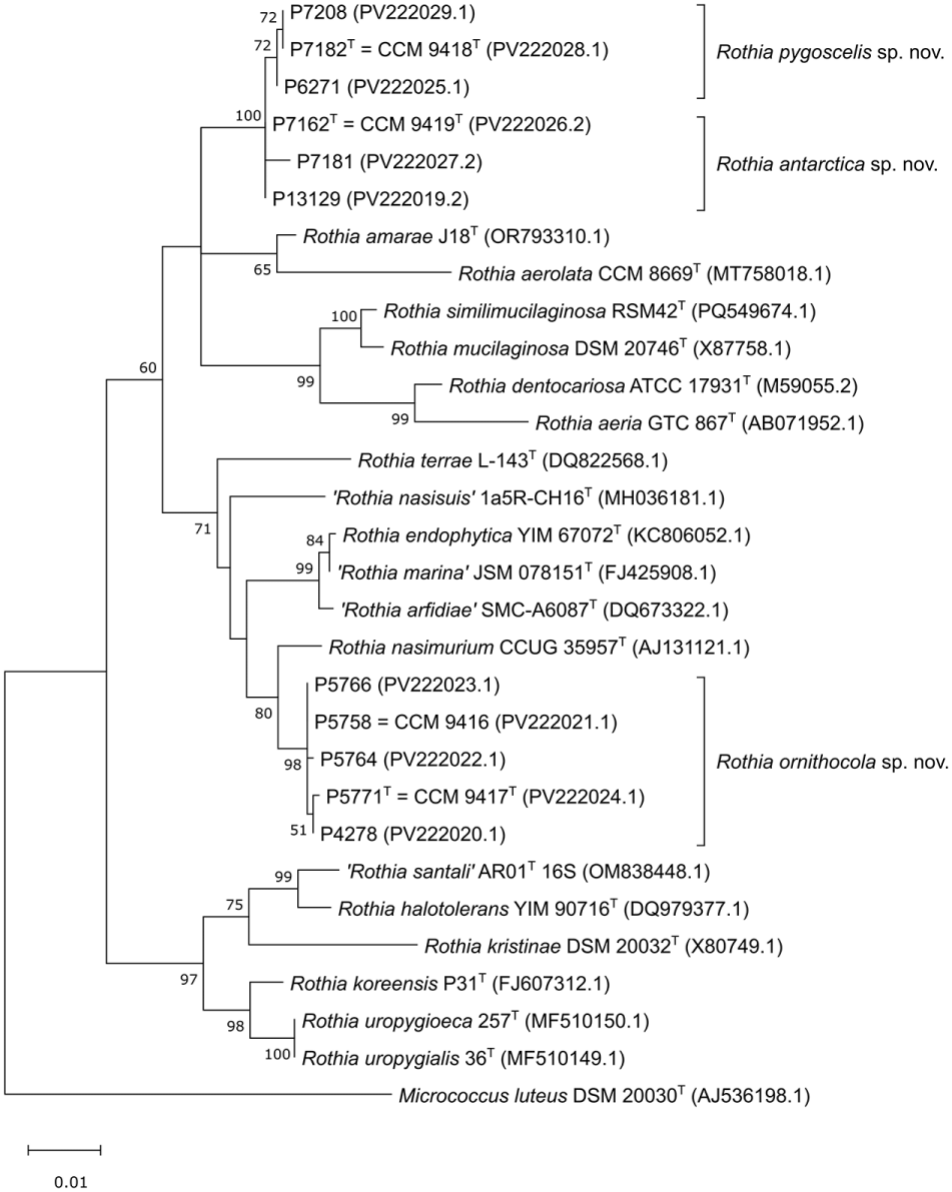
Maximum Likelihood phylogenetic tree based on 16S rRNA sequences showing the position of the studied strains within the genus *Rothia*. Numbers at nodes represent bootstrap values >50%. The final dataset comprised 1298 positions. *Micrococcus luteus* DSM 20030^T^ was used as an outgroup. Bar, 0.01 substitutions per nucleotide position.

Phylogenetic reconstruction based on 16S rRNA sequences separated the studied strains into two distinct lineages (Fig. 2). Strains P4278, P5758, P5764, P5766, and P5771^T^ clustered with *R. endophytica* and *R. nasimurium*, whereas the remaining six strains (P6271, P7162^T^, P7181, P7182^T^, P7208, and P13129) formed a cluster with *R. amarae* and *R. aerolata*. All three inference methods (Maximum Likelihood, Minimum Evolution, and Neighbor-Joining) produced consistent clustering of the studied strains and their closest relatives, with only minor topological differences observed in the Maximum Likelihood tree (Fig. 2) compared with the Minimum Evolution (Supplementary Fig. S3) and Neighbor-Joining trees; only the Minimum Evolution tree is presented as supplementary material.

Taken together, the rep-PCR, 16S rRNA gene sequence similarities and phylogenetic analyses indicate that the eleven Antarctic isolates represent three novel species within the genus *Rothia*, for which we propose the names *Rothia ornithocola* sp. nov. (strains P4278, P5758 = CCM 9416, P5764, P5766, and P5771^T^ = CCM 9417^T^), *Rothia pygoscelis* sp. nov. (strains P6271, P7182^T^ = CCM 9418^T^, and P7208), and *Rothia antarctica* sp. nov. (strains P7162^T^ = CCM 9419^T^, P7181, and P13129).

### MALDI-TOF MS

MALDI-TOF MS analysis of protein signals in the 2-12 kDa mass range allowed the studied strains to be divided into three groups, clearly distinct from the reference strains analyzed alongside them (Supplementary Fig. S2). The group assignment was supported by 19 consensus signals characteristic of *R. ornithocola* sp. nov., three specific to *R. pygoscelis* sp. nov., and six to *R. antarctica* sp. nov., with nine other peaks shared between the latter two. However, no signals were shared by all 11 studied strains. None of the 11 strains showed significant similarity to the entries in the Biotyper database (version 10.0, 9468 entries). Upon addition of the three type strain spectra, the remaining eight strains were correctly identified at the species level with high confidence (log score > 2.000), and none of the 38 strains representing the nine *Rothia* species already in the database were misassigned to the newly added entries. These results confirm the suitability of MALDI MS for the routine species-level identification of *Rothia*, as suggested previously (Zdziarski et al. 2022). List of signals is attached as Supplementary Table S2.

### Genome sequencing and assembly

The newly assembled genomes' lengths span approximately from 1.85 to 2.41 Mbp, with sequencing coverage ranging from 15× to 148×. The individual genomes contain between 1662 and 2174 predicted open reading frames (ORFs), the majority corresponding to protein-coding sequences (CDSs), alongside a small number of pseudogenes. The estimate of completeness of the assemblies ranges from 99.81 to 100% and the contamination estimate ranges from 0% to 0.03%, meeting the minimal standards for the use of genome data for the taxonomy of prokaryotes (Riesco and Trujillo 2024). Notably, *R. ornithocola* sp. nov. differs from *R. pygoscelis* sp. nov. and *R. antarctica* sp. nov. in genome size, GC content, and gene count, while the latter two species exhibit very similar genomic features, confirming their close phylogenetic relationship. Key genomic features are summarized in Supplementary Table S4.

### Comparative pan-genome analysis

**Figure 3:**
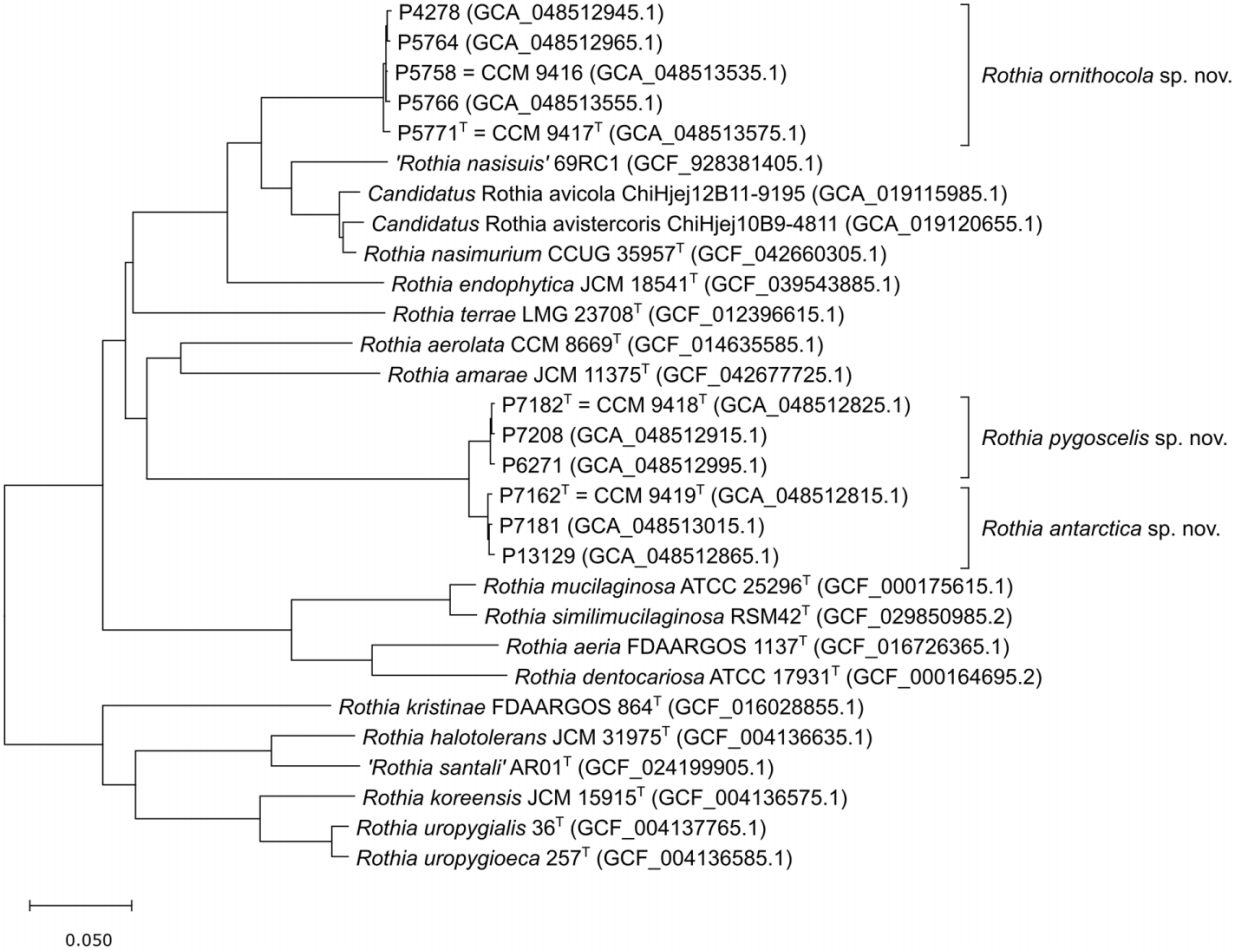
Phylogenomic tree based on concatenated sequences of 473 core genes from representative *Rothia* genomes. The tree was calculated using the BPGA (v1.3.0), reconstructed using the neighbour-joining method, and visualized with MEGA11.

Pan-genome analysis, including 18 reference *Rothia* genomes retrieved from NCBI GenBank (listed in Supplementary Table S3) and the 11 newly assembled genomes, identified 473 core genes shared across the genus. The phylogenomic tree (Fig. 3) constructed from concatenated sequences of core genes revealed that strains presented in this study formed three well-distinguished clusters corresponding to the three proposed novel species.

### Average nucleotide identity and digital DNA–DNA hybridization

**Figure 4:**
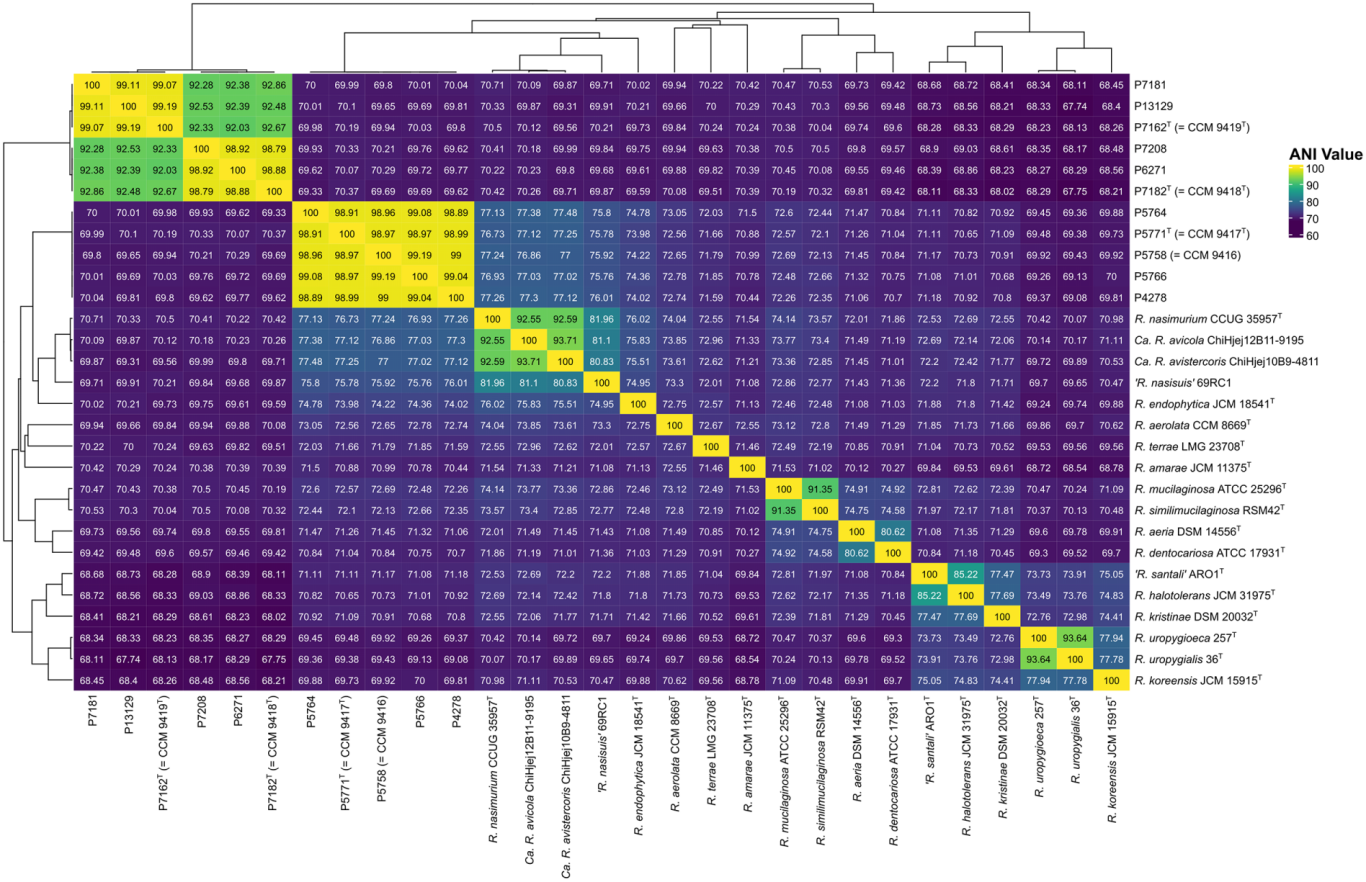
Heatmap representation of ANI values (%) calculated with OrthoANIu between the studied Antarctic strains and the 18 reference *Rothia* genomes.

The obtained ANI values supported the presence of three distinct lineages within the Antarctic strains (Fig. 4) as suggested by the phylogenomic analysis. The ANI values observed within the three strain clusters are well above the recommended 95%–96% threshold for species delineation (Richter and Rosselló-Móra 2009, Chun et al. 2018), while the ANI values between the individual clusters and reference genomes are below the threshold. This further confirms the delineation of the three novel *Rothia* species and a high degree of genomic similarity between *R. pygoscelis* sp. nov. and *R. antarctica* sp. nov. The dDDH values within *R. ornithocola* sp. nov., *R. pygoscelis* sp. nov. and *R. antarctica* sp. nov. were 90.8%–93.3%, 89.6%–90.7%, and 92.3%–93.1%, respectively. In contrast, dDDH values between *R. pygoscelis* sp. nov. and *R. antarctica* sp. nov. were below 46.5%, and values between either of these species and *R. ornithocola* sp. nov. were below 22.5%. Comparisons with other members of the genus *Rothia* yielded dDDH values below 27%. These results clearly support the separation of the studied strains into three distinct genomic clusters, each representing a novel species, as the generally accepted threshold for species delineation is 70% (Meier-Kolthoff et al. 2013, Chun et al. 2018).

### Phenotypic characterization

The colonies of all studied strains grown on TSA at 30°C were creamy white, raised with a smooth surface and entire margin. After prolonged cultivation, colonies may develop a warty surface, retaining their glossy surface, similarly to other *Rothia* species (Austin 2015). The cells are spherical, 0.5–1.3 μm in diameter and Gram-variable.

The phenotypic tests allow distinguishing the three novel species from each other and the reference strains. The differentiating traits are listed in Table 2. The full results of the phenotypic characterization can be found below in the species protologues.

**Table 2:** Phenotypic characteristics distinguishing the novel *Rothia* species and reference strains.

	*R. ornithocola* sp. nov.	*R. pygoscelis* sp. nov.	*R. antarctica* sp. nov.	*R. endophytica* CCM 9215^T^	*R. nasimurium* CCM 9216^T^	*R. amarae* CCM 9402^T^
**Growth in**						
15°C	–	–	–	w	+	+
20°C	+	w	–	+	+	+
40°C	+	var	–	–	–	+
8% NaCl	+	var	–	+	+	+
9% NaCl	+	var	–	w	+	+
Gelatine hydrolysis	var	var	+	+	w	–
β-galactosidase production (ONPG)	–	–	–	+	+	–
Aesculin hydrolysis	–	–	–	+	+	w
Nitrate reduction	+	–	–	+	+	+
Tyrosine hydrolysis	+	–	–	+	+	+
**API ZYM**						
Alkaline phosphatase	–	–	+	–	–	+
Esterase (C4)	var	–	–	–	+	w
Cystine arylamidase	–	w	–	–	+	+
b-glucuronidase	–	–	–	–	–	+
b-glucosidase	–	–	–	–	–	+
**STAPHYtest 24**						
ß-glucosidase	–	–	–	–	w	+
Trehalose	+	var	–	+	+	+
Mannitol	–	–	–	–	–	+
Mannose	+	+	+	–	+	+
Fructose	var	–	var	+	+	+
Acetoin production	+	+	var	–	–	+

All the results were obtained in this study. +, Positive; w, weakly positive; −, negative; var, variable.

### Susceptibility to antibiotics

All studied strains, including the reference strains, were resistant to penicillin G. Some strains also exhibited resistance to ampicillin, cefepime, and imipenem, and several strains showed intermediate resistance to ciprofloxacin (Table 3). When compared to the reference strains, the Antarctic strains show susceptibility to a broader range of antimicrobials. Interestingly, genomic analysis (see below) did not reveal the presence of resistance genes in their genomes, suggesting the existence of yet unknown genetic background of the observed resistance.

**Table 3:** Results of susceptibility to antimicrobials testing of studied *Rothia* strains and reference strains.

	*R. ornithocola* sp. nov.	*R. pygoscelis* sp. nov.	*R. antarctica* sp. nov.	*R. endophytica* CCM 9215^T^	*R. nasimurium* CCM 9216^T^	*R. amarae* CCM 9402^T^
Ampicillin	R (100)	R (100)	Var (33)	R	R	R
Cefepime	Var (60)	Var (67)	S	S	S	R
Ciprofloxacin	I (100)	Var (33 I)	Var (33 I)	I	I	I
Clindamycin	S	S	S	R	R	R
Erythromycin	S	S	S	R	R	S
Moxifloxacin	S	S	S	S	S	S
Gentamicin	S	S	S	S	S	S
Chloramphenicol	S	S	S	R	R	S
Linezolid	S	S	S	S	S	S
Imipenem	R (100)	Var (67)	Var (67)	R	R	R
Penicillin G	R (100)	R (100)	R (100)	R	R	R
Rifampicin	S	S	S	R	R	S
Cotrimoxazole	S	S	S	S	S	S
Tetracycline	S	S	S	S	S	S
Vancomycin	S	S	S	S	S	S

The percentage of studied strains resistant or intermediately resistant to the tested antibiotic is given in parentheses. S, susceptible, I, intermediate, R, resistant, Var, variable.

As the breakpoint values were taken from guidelines for various bacterial genera, the inhibition zone diameters together with the breakpoints used are shown in Supplementary Table S5.

### Fatty acid methyl esters analysis

The most abundant cellular fatty acids were iso- and anteiso-methyl branched fatty acids, with C_15:0_ anteiso being predominant across all studied strains (mean of 75.3%) as well as the reference strains. This is consistent with the characteristics previously described for the three reference species and the genus *Rothia* in general (Collins et al. 2000, Fan et al. 2002, Xiong et al. 2013, Austin 2015).

*Rothia ornithocola* sp. nov. strains showed consistent fatty acid profiles. In comparison to the closest relative *R. nasimurium* CCM 9216^T^, the studied strains contained higher amounts of C_15:0_ anteiso (mean of 83.4%) and lower amounts of iso- branched fatty acids, C_14:0_ and C_16:0_. Fatty acid profiles of *R. pygoscelis* sp. nov. and *R. antarctica* sp. nov. were similar for all isolates. The only notable difference was a slightly lower amount of C_15:0_ iso fatty acid in strains of *R. pygoscelis* sp. nov. compared to *R. antarctica* sp. nov. isolates. In comparison to the closest relative *R. endophytica* CCM 9215^T^ and *R. amarae* CCM 9402^T^, both novel species revealed higher amounts of shorter iso- and anteiso-branched fatty acids (C_13:0_ anteiso, C_14:0_ iso and C_15:0_ iso) and lower amounts of C_17:0_ anteiso.

Detailed cellular fatty acid composition of the studied *Rothia* isolates and the reference strains is presented in Supplementary Table S6.

### Functional genomic analysis

Prophage analysis revealed no intact, questionable, or incomplete prophage elements in any of the genomes. This absence of foreign genetic material may indicate the presence of an active bacterial immune defence. Consistently, CRISPR arrays, constituting an adaptive immune system in bacteria, were detected in all strains except P5764 and those with at least medium confidence according to CRISPRdetect are available in Supplementary Table S8. R-M systems were identified in all genomes except P7182^T^ (= CCM 9418^T^), although their composition varied substantially (Supplementary Table S9). Type I systems, found predominantly in *R. ornithocola* sp. nov., were mostly complete and displayed the canonical three–subunit organization (R-M-S); exceptions include P5758 (= CCM 9416) and P5766, where the M and R subunits were missing, respectively. Type II system consisting of a two–gene RM arrangement, was detected across all groups, while type III systems were found exclusively in *R. antarctica* sp. nov. Additionally, several genomes encoded Type IIG systems, where restriction and methylation functions are fused into a single polypeptide, and a subset of strains carried Type IV single–gene nucleases.

Screening against the CARD database using RGI did not identify any antibiotic resistance genes with >50% identity in the analyzed genomes. This contrasts with the phenotypic resistance observed for several antibiotics and suggests that the underlying resistance determinants may differ from currently characterized resistance genes. The lack of identifiable matches highlights current gaps in genomic annotation of resistance mechanisms in understudied bacterial taxa (Centner et al. 2026).

KEGG-based functional annotation revealed diverse metabolic capabilities in the genomes of all three novel species. Most assigned KEGG Orthologs (KOs) were associated with carbohydrate metabolism, followed by amino acid metabolism and metabolism of cofactors and vitamins, a distribution that is typical of heterotrophic bacterial genomes. This corresponds to the general view of *Rothia* species as metabolically versatile bacteria capable of both synthesizing and utilizing diverse carbohydrates and amino acids (Lim et al. 2013, Lu et al. 2025). *Rothia pygoscelis* sp. nov. and *R. antarctica* sp. nov. shared a largely overlapping set of complete KEGG modules, whereas several differences distinguished these two species from *R. ornithocola* sp. nov.

Both *R. pygoscelis* sp. nov. and *R. antarctica* sp. nov. encoded a complete Leloir pathway for galactose degradation—galM (K01785), galK (K00849), galT (K00965), and galE (K01784), while all *R. ornithocola* sp. nov. genomes lacked galM (K01785) and galT (K00965). Consistent with these genomic predictions, galactose utilization was detected in several penguin-associated strains (P7162^T^ = CCM 9419^T^, P7182^T^ = CCM 9418^T^, P7208) but was not observed in any *R. ornithocola* sp. nov. strain. None of the 11 genomes encoded a β-galactosidase (K01190) or a lactose permease (K02532), in agreement with the uniformly negative ONPG, API ZYM β-galactosidase, α-galactosidase, and lactose acidification results across all strains. Because the strains likely cannot cleave galactose from glycoconjugates themselves, the galactose they utilize needs to be available as a free monosaccharide. In oral and mucosal niches, free galactose is typically released by exoglycosidases of co-resident microbes from terminal Gal residues of host mucin O-glycans and other host glycoproteins (Tailford et al. 2015). *Rothia ornithocola* sp. nov. possessed complete biosynthetic pathways for lysine, ornithine, arginine and proline, which were incomplete in the other two species. Loss of amino acid biosynthetic pathways can be an evolutionary strategy in host-associated microbial environments where amino acids are readily available, conferring a selective advantage through reduced metabolic burden (Morris et al. 2012, Ramoneda et al. 2023).

A pronounced species-level signal was observed for nitrate reduction: all *R. ornithocola* sp. nov. genomes encoded three membrane-bound nitrate reductase subunits narG (K00370), narH (K00371), narI (K00374), whereas these KOs were absent from all *R. pygoscelis* sp. nov. and *R. antarctica* sp. nov. genomes, mirroring the positive nitrate reduction phenotype observed only in *R. ornithocola* sp. nov. Respiratory nitrate reduction enables anaerobic energy conservation using nitrate as a terminal electron acceptor and is a recognized adaptation to the hypoxic, nitrate-containing intestinal lumen (Moreno-Vivián et al. 1999).

Furthermore, several genes associated with the biosynthesis of siderophore group nonribosomal peptides were identified in all 11 genomes but were absent from the three reference species genomes. This finding prompted further investigation of the biosynthetic potential of the Antarctic bird-associated strains.

Functional annotation of CDSs using COGs classification assigned the majority of genes to specific functional categories (Supplementary Table S7). However, 14.8%–18% of genes were assigned to category S, function unknown, and additional 4.5%–23% of genes were not assigned to any of the COGs. This suggests that a notable portion of the predicted genes serve functions that remain poorly characterized, and another major portion of the genes significantly differs from the database entries. Such proportions are not unusual for a relatively understudied genus, although the limited representation of host-associated lineages from the Antarctic region may also contribute to this pattern. The functional profiles were highly similar across all genomes, with minimal differences in category distribution, supporting the close phylogenetic relationship among the strains; no clear distinctions were observed between *R. ornithocola* sp. nov., *R. pygoscelis* sp. nov., and *R. antarctica* sp. nov. (Supplementary Fig. S4).

**Figure 5:**
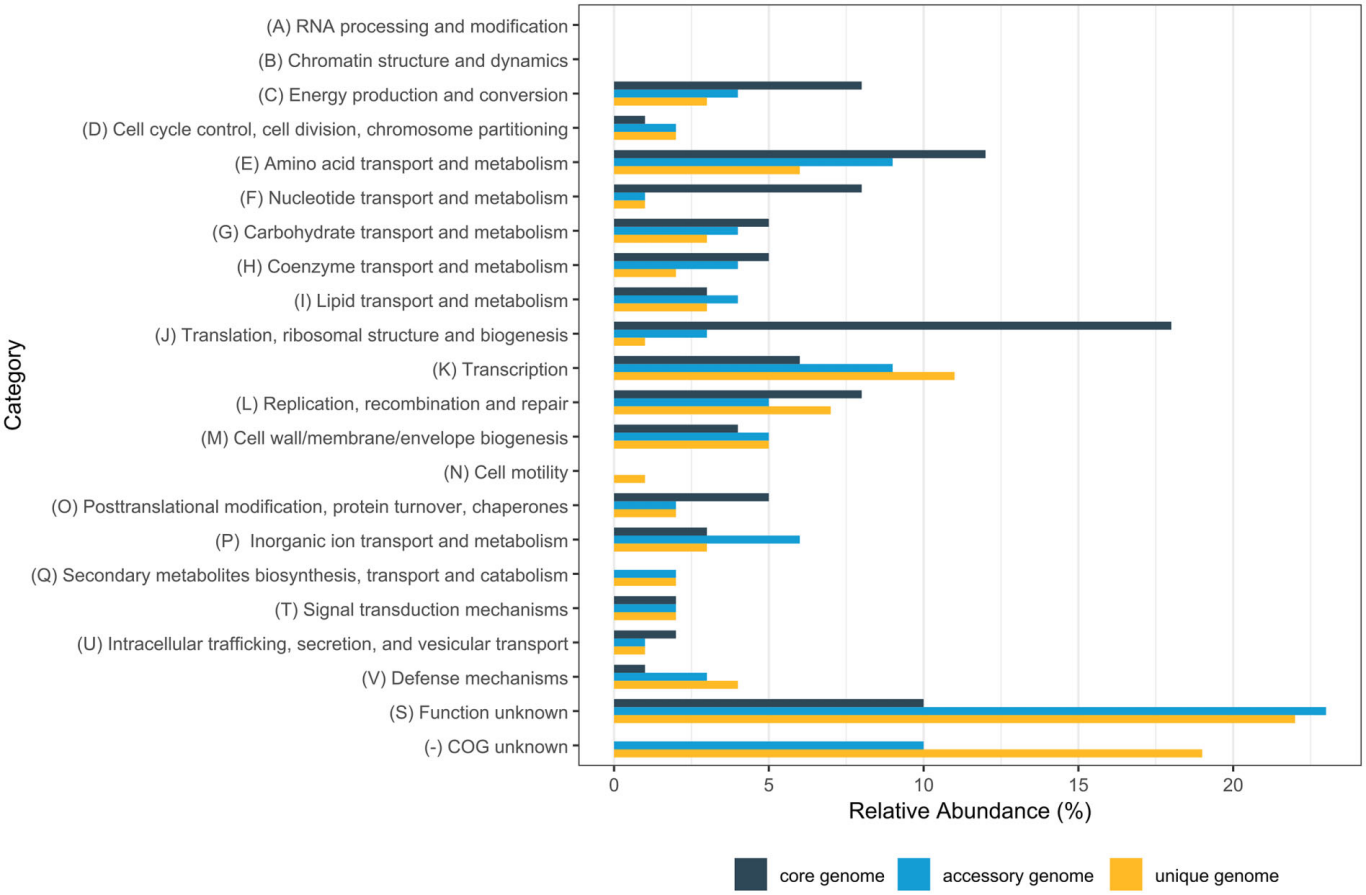
Relative abundance (%) of genes in *Rothia* pan-genome assigned to each Clusters of Orthologous Groups (COGs) functional category in the core (dark blue), accessory (light blue), and unique (yellow) genome fractions. Pan-genome analysis of 29 *Rothia* genomes was performed using BPGA v1.3.0 with a 50% amino acid identity cut-off.

Comparison of COG category distributions across the core, accessory, and unique fractions of the *Rothia* pan-genome revealed clear functional partitioning between conserved and variable portions of the genome (Fig. 5). The core genome was strongly enriched in categories associated with essential cellular functions, most notably translation, ribosomal structure, and biogenesis (J), as well as nucleotide transport and metabolism (F), energy production and conversion (C), amino acid transport and metabolism (E), and posttranslational modification, protein turnover and chaperones (O). This distribution is consistent with the expected conservation of housekeeping machinery across the genus (Brockhurst et al. 2019).

In contrast, the accessory and unique fractions were dominated by genes of unknown function (categories S and -), which together accounted for approximately one third of the accessory genome and over 40% of the unique genome, compared with only ∼10% in the core. Transcription (K) was also proportionally more abundant in the accessory and unique fractions, as were inorganic ion transport and metabolism (P), defence mechanisms (V), cell wall, membrane and envelope biogenesis (M), and secondary metabolites biosynthesis, transport, and catabolism (Q). This pattern indicates that variation between *Rothia* strains and species is driven predominantly by regulatory genes, lineage-specific defence and surface systems, and a substantial reservoir of functionally uncharacterized genes, consistent with a typical pan-genome architecture in which conserved housekeeping functions are complemented by a flexible component associated with niche adaptation and strain-specific traits (Vernikos et al. 2015, Brockhurst et al. 2019).

### Biosynthetic gene cluster analysis

**Figure 6:**
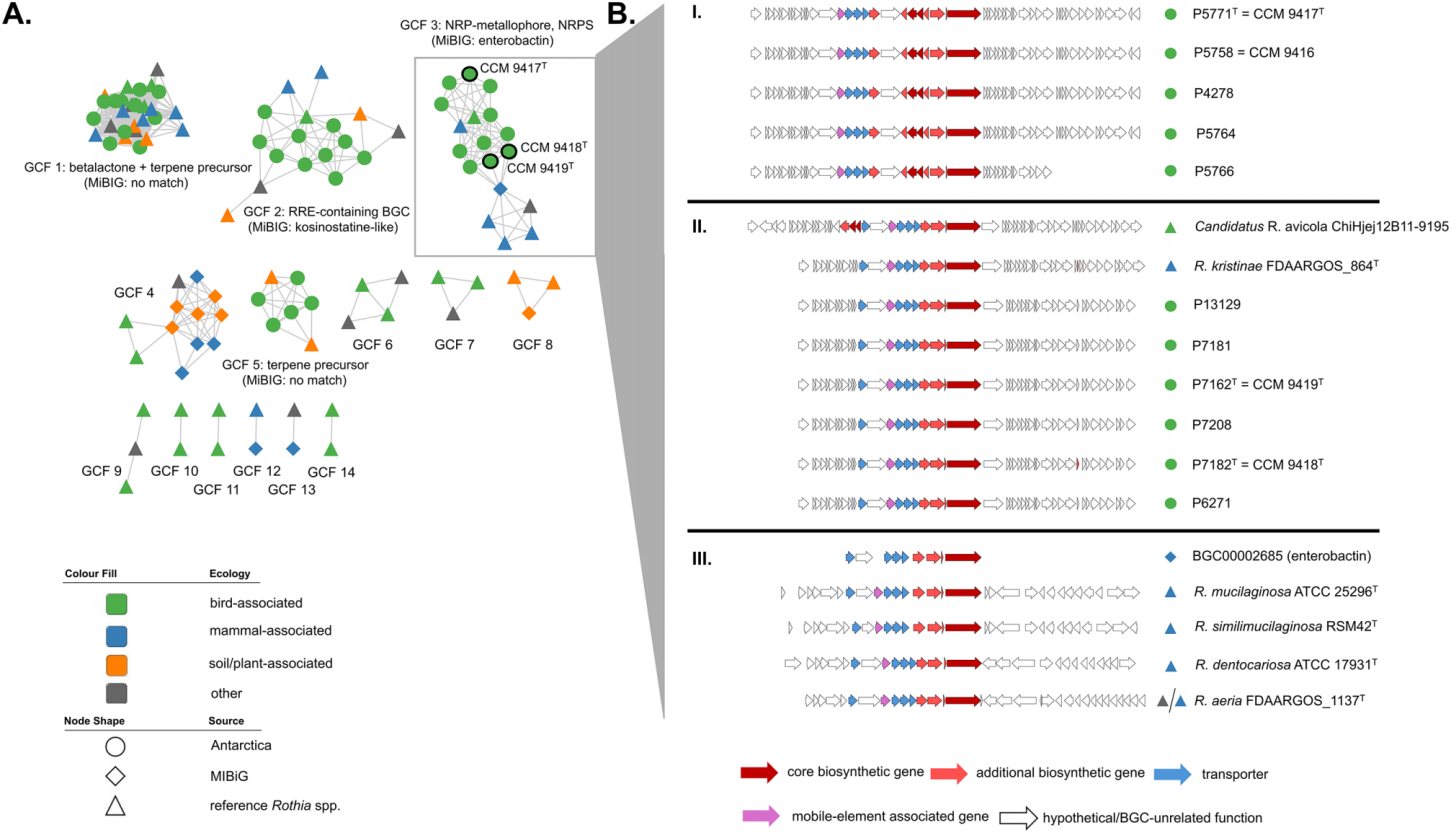
BiG-SCAPE (v2.0.0) sequence similarity network (SSN) (c = 0.4) of *Rothia* spp. visualized in Cytoscape (v3.10.3). (A) SSN (*c* = 0.4) of *Rothia* spp. Each node represents a BGC identified by antiSMASH (v8.0.0) and the length of edges represents their genetic relatedness calculated using BiG-SCAPE. Colours indicate ecological source. Node shapes correspond to strain groups, including Antarctic *Rothia* strains, reference *Rothia* spp. and MiBIG v3.1 reference BGCs. All identified BGCs formed gene cluster families (GCFs); therefore, no singleton clusters are shown. (B) Identified pathways representing three subgroups (I to III) within the GCF 3 are shown. Gene colouring according to antiSMASH annotations.

To explore the specialized metabolite biosynthetic potential of Antarctic bird-associated *Rothia* strains, genomes were analyzed using antiSMASH. All Antarctic bird-associated *Rothia* strains contained 2–5 predicted biosynthetic gene clusters (BGCs) per genome, including clusters potentially encoding terpenes, siderophores, and other specialized metabolites (Supplementary Table S10). To assess the diversity and relatedness of BGCs within the genus *Rothia* and to evaluate their broader ecological and evolutionary relationships, BGCs detected in both Antarctic and reference *Rothia* genomes were used for similarity network analyses which were performed using BiG-SCAPE at both the default cut-off (c = 0.5; Supplementary Fig. S5) and a stricter cut-off (c = 0.4; Fig. 6). While the relaxed threshold enabled comparison with more distantly related reference BGCs from the MIBiG database, the stricter threshold provided improved resolution of BGC relationships within the *Rothia* genus.

Similarity network analysis using a stringent BiG-SCAPE cut-off (c = 0.4) revealed clear ecological and taxonomic structuring of several gene cluster families (GCFs) (Fig. 6). The four major GCFs represented by Antarctic strains comprised between 7 and 28 BGCs (GCF 1–3 and GCF 5) and were predominantly associated with terpene, terpene/β-lactone hybrid, and restriction recognition element (RRE)-containing pathways (Fig. 6A). Most major GCFs were composed of strains from multiple ecological backgrounds, including bird-associated, animal-associated, soil/plant-associated, and other environmental sources. In contrast, GCF 3 displayed a markedly different distribution pattern and consisted almost exclusively of animal- and bird-associated strains. This GCF comprised BGCs encoding nonribosomal peptide synthetases (NRPS) and NRPS/nonribosomal peptide-metallophore (NRP-metallophore) hybrids and clustered with the MIBiG reference BGC0002685.2 encoding enterobactin biosynthesis in *R. mucilaginosa* ATCC 25296^T^ (Uranga et al. 2020). The only non-host-associated exception within this GCF was NRPS cluster from *Rothia aeria* FDAARGOS_1137^T^, originally isolated from air samples collected aboard the Mir space station (Li et al. 2004). However, *R. aeria* is recognized as a common oral commensal in humans (Wei et al. 2020, Mazurel et al. 2023), suggesting that its detection in the space station environment likely reflects human-associated microbial dispersal rather than adaptation to a free-living environmental niche. Analysis using a more relaxed clustering threshold (c = 0.5; Supplementary Fig. S5) resulted in only limited expansion of this GCF. In addition to the already identified enterobactin-like clusters, the relaxed network incorporated the enterobactin-associated BGC from the bird-associated species *R. uropygioeca* 257^T^. The only additional non-*Rothia* connection was represented by BGC0000615 from marine *Nocardiopsis* sp., annotated in MIBiG as a thiopeptide RiPP pathway. However, this association was limited and likely reflects partial similarity within shared biosynthetic domains rather than close overall pathway relatedness.

Further inspection of GCF 3 revealed additional internal structure associated with differences in cluster architecture (Fig. 6B). Based on BiG-SCAPE family subdivision patterns and comparative organization of biosynthetic genes, the enterobactin-like GCF could be further resolved into three distinct subgroups (I–III). Subgroup I consisted exclusively of Antarctic bird-associated strains (*R. ornithocola* sp. nov.) and was distinguished by the presence of NRPS/nonribosomal peptide-metallophore (NRP-metallophore) hybrid architecture. A related NRPS/NRP-metallophore-associated BGC with distinct cluster organization was also identified in the bird-associated strain "*Candidatus* R. avicola" ChiHjej12B11-9195, which formed an intermediate lineage positioned close to subgroup I in the phylogenetic reconstruction. Subgroup II comprised six Antarctic BGCs originating from two newly described Antarctic species (*R. antarctica* sp. nov. and *R. pygoscelis* sp. nov.). In contrast, subgroup III contained the reference enterobactin BGC from *R. mucilaginosa* ATCC 25296^T^ together with additional non-Antarctic *Rothia* strains, representing lineage of predominantly human- and mammal-associated enterobactin-like BGCs within the network.

**Figure 7:**
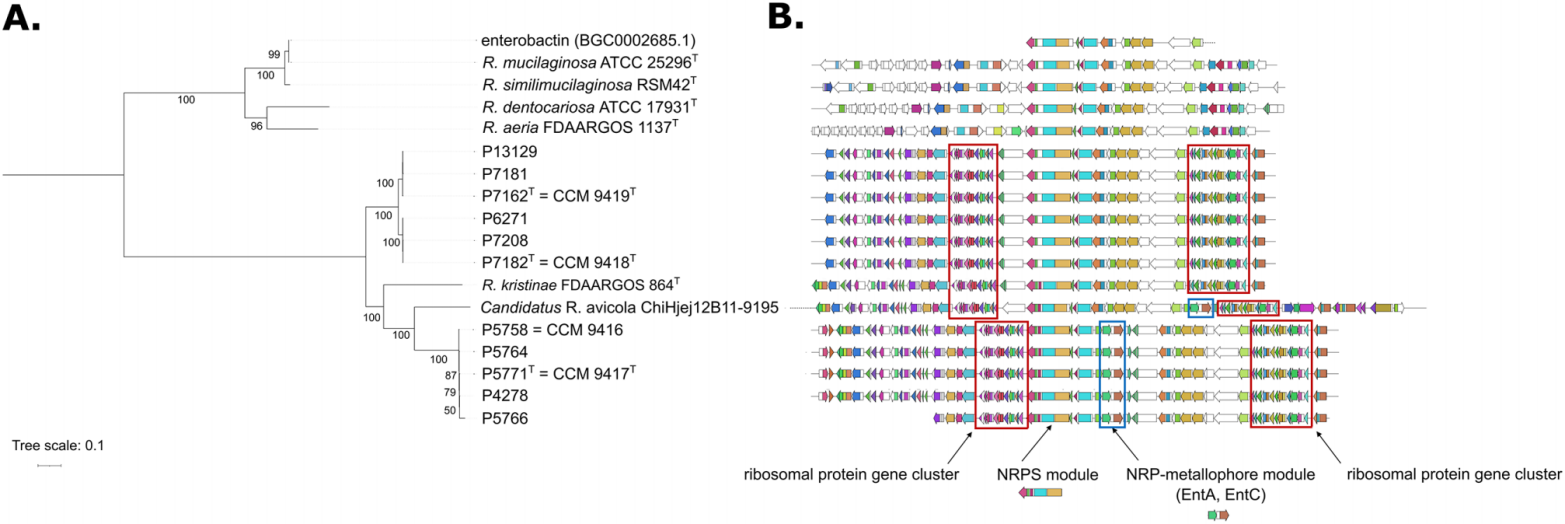
Maximum-likelihood phylogeny and comparative CORASON analysis of enterobactin-like biosynthetic gene clusters (BGCs) identified within GCF 3. The phylogeny was reconstructed from BiG-SCAPE GCF alignments using 1000 bootstrap replicates and midpoint-rooted for visualization. Conserved core biosynthetic regions corresponding to the enterobactin-associated NRPS module are indicated. Coloured boxes highlight metallophore modules and conserved flanking ribosomal protein gene clusters shared by subgroup I and II lineages. The scale bar represents the number of nucleotide substitutions per site.

Maximum-likelihood phylogenetic reconstruction (Fig. 7A) and comparative analysis of cluster architecture using CORASON (Fig. 7B) confirmed architectural differences among the three enterobactin-like subgroups while preserving the conserved core NRPS-associated biosynthetic region. Interestingly, the Antarctic-specific subgroup I containing additional NRP-metallophore-associated features was recovered exclusively from skua- and gull-associated isolates (and unknown bird feathers/faeces), whereas penguin-associated strains clustered within subgroup II revealing more canonical enterobactin-like pathways related to *R. mucilaginosa*. Although the number of available genomes remains limited, this pattern suggests that diversification of enterobactin-like BGCs in Antarctic *Rothia* may partly reflect adaptation to distinct avian host-associated ecological niches. In addition to differences in biosynthetic gene composition, subgroup I and II enterobactin-like BGCs shared a conserved surrounding genomic context distinct from that observed in *R. mucilaginosa* ATCC 25296^T^, including conserved flanking ribosomal protein gene clusters absent from subgroup III-associated loci (Fig. 7B). Together, these observations support progressive diversification of enterobactin-like biosynthetic pathways within host-associated *Rothia* lineages, consistent with lineage-associated evolution of specialized metabolism reported in other bacterial groups (Chase et al. 2021).

Iron acquisition represents a major selective pressure in host-associated environments, where vertebrate nutritional immunity strongly limits iron availability on mucosal surfaces (Ellermann and Arthur 2017, Kramer et al. 2020). High-affinity siderophore systems are therefore common colonization and persistence factors among host-associated bacteria, where they play roles not only in iron acquisition, but also in stress tolerance, microbial signalling and interspecies competition within host microbiota (Brickman and Armstrong 2009, Schalk et al. 2011, Kramer et al. 2020). Consistent with this ecological role, members of the genus *Rothia* are increasingly recognized as specialized mucosal colonizers of oral and respiratory environments, where iron availability is strongly restricted by host nutritional immunity (Rigauts et al. 2022, Natalini et al. 2023, Choi et al. 2025). Enterobactin production has previously been demonstrated in *R. mucilaginosa* in suboptimal growth conditions and proposed to contribute to host colonization (Uranga et al. 2020). The strong enrichment of enterobactin-like BGCs among bird- and animal-associated *Rothia* lineages observed in this study therefore supports the hypothesis that siderophore biosynthesis represents a conserved ecological strategy facilitating persistence within iron-limited host-associated niches.

### Siderophore assays

**Figure 8:**
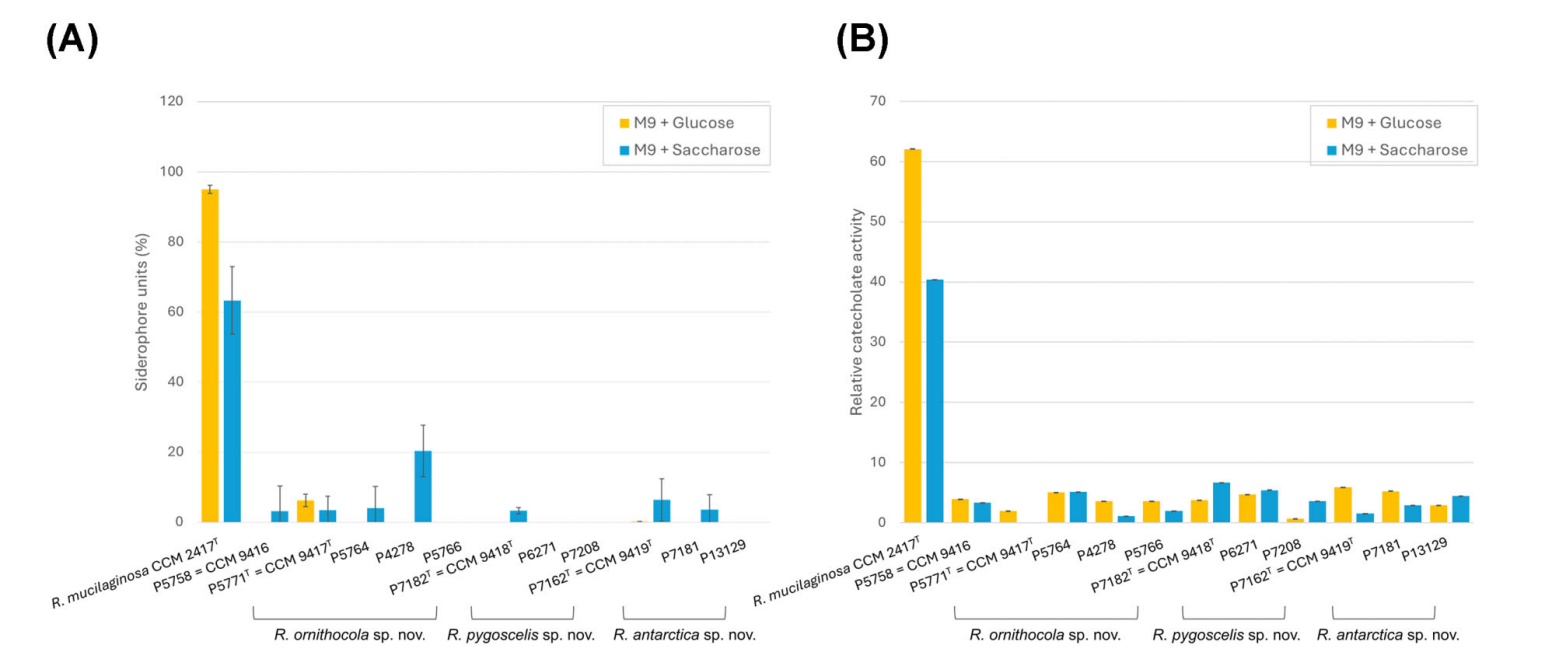
Detection of siderophore and catecholate-like activity in Antarctic *Rothia* strains cultivated in iron-limited M9 minimal medium supplemented with glucose or saccharose. (A) Liquid Chrome Azurol S (CAS) assay showing siderophore units (%) after 3 days of cultivation. (B) Arnow's assay showing relative catecholate activity expressed as percentage of 100 µM catechol control. Values represent mean ± SD from three biological replicates. *Rothia mucilaginosa* CCM 2417^T^ was used as a positive control. Species affiliations of Antarctic isolates are indicated below the *x*-axis.

Siderophore production was detected only in the positive control strain, *R. mucilaginosa* CCM 2417^T^, which produced a clear positive reaction in the liquid CAS assay after three days of cultivation and a moderately positive signal in the Arnow's assay, consistent with catecholate-type siderophore production (Arnow 1937, Gomes et al. 2024) (Fig. 8A). In contrast, none of the Antarctic *Rothia* isolates showed clear detectable activity in either assay despite the presence of enterobactin-like BGCs in several genomes. Although strain P4278 exhibited moderately elevated CAS values under saccharose supplementation (∼20% siderophore units), no corresponding visible colour change was observed, and the strain remained negative in the Arnow's assay. These observations suggest that the spectrophotometric shift likely reflected physicochemical effects influencing the CAS assay rather than clear siderophore production (Louden et al. 2011). Furthermore, several Antarctic strains exhibited low relative catecholate activity values (∼5%) in the Arnow's assay (Fig. 8B). These signals were not accompanied by visible colour change and were therefore interpreted as negative or background-level responses. Similarly, no visible colour transition was observed for Antarctic strains in the liquid CAS assay. Later cultivation timepoints (days 5–7) showed increased variability in CAS readouts; however, these measurements were accompanied by medium acidification without corresponding CAS colour change, suggesting medium-dependent interference affecting assay stability rather than true siderophore production (Murakami et al. 2021, Gomes et al. 2024). The agar-based CAS assay could not be reliably evaluated because all tested strains, including the positive control, exhibited only limited growth under the tested conditions.

The absence of detectable siderophore activity in Antarctic *Rothia* strains, despite cultivation in iron-limited M9 medium, suggests that these pathways may not be induced by iron limitation alone. Host-associated conditions in the native environment are likely substantially more complex than standard laboratory cultivation and may involve specific interactions with host tissues, competing microbiota, nutritional immunity, or temperature-dependent regulatory cues that influence pathway activation (Hood and Skaar 2012, Kramer et al. 2020, Song et al. 2024, Amiri et al. 2025). Enterobactin and related catecholate siderophores are known to display environmentally responsive expression patterns influenced not only by iron limitation, but also by temperature and host-associated conditions in several bacterial systems (Valdebenito et al. 2006, Dumas et al. 2013, Li H et al. 2015, Gasser et al. 2016, Amiri et al. 2025). These findings therefore highlight the importance of investigating the regulatory mechanisms controlling siderophore biosynthesis and the environmental signals required for production of the final metabolite and its potential ecological role in bird-associated *Rothia* populations.

### Environmental dispersal

For *R. ornithocola* sp. nov., Branchwater returned several hits with containment values up to 0.32 and cANI values up to 0.95 (Supplementary Table S11). Although these matches remain just below the 95% ANI threshold for species delineation (Chun et al. 2018) and therefore do not identify conspecific genomes in the public metagenomic record, the highest-ranked hits originated from a coherent set of bird-associated samples, including ambient outdoor air from an avian park, swan and chicken stool, goose intestinal content, and duck slaughterhouse wastewater. This ecological consistency is in line with the previously reported occurrence of *Rothia* species across diverse avian hosts, including chickens, geese, woodpeckers, ostriches, and hummingbirds (Braun et al. 2018, Abolnik et al. 2021, Herder et al. 2021, Kang et al. 2022, Zhang et al. 2022), and indicates that close relatives of *R. ornithocola* sp. nov. are likely to be present in bird-associated environments worldwide, while the species itself remains absent from publicly available metagenomic surveys. A similar use of Branchwater to detect the global distribution of organisms originally isolated from polar habitats has been demonstrated for Antarctic cyanobacteria (Lumian et al. 2024), supporting the use of k-mer-based queries to extend ecological inference beyond the original isolation sites.

For *R. pygoscelis* sp. nov. and *R. antarctica* sp. nov., the top-ranked hits did not exceed 0.11 containment, with cANI values reaching at most 0.90 (Supplementary Table S11). Across the full result sets, containment values clustered tightly at the lower limit of Branchwater reliability and were distributed at near-uniform levels across taxonomically and ecologically unrelated metagenomes—including wetland, soil, marine sediment, estuary, glacier, and hydrothermal-vent samples spanning every populated continent and both polar regions. The absence of any biome- or location-specific enrichment, combined with cANI values well below the species delineation threshold (Chun et al. 2018), indicates that these matches reflect sparse k-mer overlap at the limit of detection rather than the presence of close relatives. Notably, although the two species were isolated from the oral cavities of penguins, no penguin- or other host-associated metagenomes appeared among the top hits, which is consistent with the broader scarcity of Antarctic avian microbiome data in public repositories (Kong et al. 2023, Goldenberg-Barbosa et al. 2025). Together, these results indicate that *R. pygoscelis* sp. nov. and *R. antarctica* sp. nov., along with their nearest relatives, represent currently undersampled lineages within the genus *Rothia* and are not yet captured by publicly available metagenomic surveys.

In the current release (R11-RS232, April 2026) of the Genome Taxonomy Database (GTDB; https://gtdb.ecogenomic.org/) (Parks et al. 2026), the genomes of all 11 Antarctic strains were classified into three separate placeholder species clusters: *Rothia* sp048512945 (corresponding to *R. ornithocola* sp. nov.), *Rothia* sp048512825 (*R. pygoscelis* sp. nov.), and *Rothia* sp048512815 (*R. antarctica* sp. nov.). Each of these clusters contained exclusively the genomes generated in the present study, with no other publicly available *Rothia* genome (isolate or MAG) meeting the default GTDB species-clustering criteria of ≥95% ANI and ≥50% orthologous regions shared with the species representative (Parks et al. 2020). This independent, genome-based classification corroborates the conclusion drawn from the Branchwater analysis, namely that close relatives of *R. ornithocola* sp. nov., *R. pygoscelis* sp. nov., and *R. antarctica* sp. nov. are currently absent from public sequence repositories.

## Conclusions

We have isolated 11 strains of Gram-variable cocci from penguins and other Antarctic birds and bird-associated material as a part of a long-term sampling campaign in the Antarctic Peninsula region. The strains were clustered using rep-PCR fingerprinting and consequently identified as members of the genus *Rothia* based on 16S rRNA gene sequences. Whole-genome sequence analyses separated the strains into three groups and confirmed they constitute three novel species within the genus *Rothia*. Further genomic analyses identified various BGCs in all studied genomes, potentially encoding terpenes, siderophores, including variants of enterobactin, and other bioactive compounds. A range of phenotypic data was collected to supplement the genomic analyses and allowed for differentiation of the studied strains from the reference *Rothia* type strains. Based on the obtained results, we conclude that these 11 isolates represent three novel species within the genus *Rothia*, and their descriptions are given below.

### *Rothia ornithocola* sp. nov.

or.ni.tho' co.la. Gr. masc./fem. n. *ornis*, bird; L. masc./fem. n. suff. *-cola*, dweller; from L. masc./fem. n. *incola*, dweller; N.L. fem. n. *ornithocola*, bird dweller.

Cells are Gram-variable, non-spore forming, non-motile, coccoid, 0.5–1.3 μm in diameter, varying in size, occurring in clusters. Grows aerobically. Forms raised, creamy white, smooth colonies with entire margins when cultivated on TSA at 30°C for 24 h. Grows within one day on TSA and blood agar. Grows well at 20–40°C, some strains grow weakly in 45 °C. Tolerates up to 9% NaCl (w/v); some strains tolerate 10% (w/v) NaCl. The major cellular fatty acid is C_15:0_  *anteiso*.

Positive for catalase production, reduction of nitrates and tyrosine hydrolysis. Variable for DNA and gelatin hydrolysis. Negative for production of acetoin, pyrrolidonyl arylamidase, β-galactosidase (ONPG test), lecithinase (egg-yolk reaction), and urease. Negative for Tween 80, arginine, aesculin, starch, and casein hydrolysis.

STAPHYtest 24 positive for sucrose, trehalose, maltose, and mannose acidification and acetoin production. Variable for fructose acidification. Negative for urea hydrolysis, arginine dihydrolase, ornithine decarboxylase, ß-galactosidase, ß-glucuronidase, ß-glucosidase, phosphatase, aesculin hydrolysis, N-acetyl-β-D-glucosamine, galactose, mannitol, xylose, lactose, sorbitol, ribose, cellobiose, arabinose, raffinose, and xylitol acidification.

API ZYM positive for leucine arylamidase, naphthol-AS-Bl-phosphohydrolase, and α-glucosidase. Variable for esterase (C4), esterase lipase (C8), lipase (C14), and valine arylamidase. Negative for alkaline phosphatase, cystine arylamidase, trypsin, α-chymotrypsin, acid phosphatase, α-galactosidase, β-galactosidase, β-glucuronidase, β-glucosidase, N-acetyl-β-glucosaminidase, α-mannosidase, and α-fucosidase.

The type strain, P5771^T^ (= CCM 9417^T^ = LMG 33815^T^), was isolated from skua (*Stercorarius* sp.) faeces at James Ross Island in the Antarctic Peninsula region. The genomic G+C content of the type strain is 56.5 mol%. The GenBank/EMBL/DDBJ accession number for the 16S rRNA sequence of the type strain is PV222024, and the whole-genome sequence accession number is JBLXXT000000000.

### *Rothia pygoscelis* sp. nov.

py.go.sce'lis. N.L. gen. fem. n. *pygoscelis*, of the penguin genus *Pygoscelis* from which the type strain was isolated.

Cells are Gram-variable, non-spore forming, non-motile, coccoid, 0.5–1.3 μm in diameter, varying in size, occurring in clusters. Grows aerobically. Forms raised, creamy white, smooth colonies with entire margin when cultivated on TSA at 30 °C for 48 h. Grows on TSA and blood agar. Grows at 20–37 °C, some strains tolerate up to 40 °C. Individual strains tolerate up to 7–9% NaCl (w/v). The major cellular fatty acid is C_15:0_  *anteiso*.

Positive for catalase production, DNA and gelatine hydrolysis. Negative for production of acetoin, pyrrolidonyl arylamidase, β-galactosidase (ONPG test), lecithinase (egg-yolk reaction), and urease. Negative for Tween 80, arginine, aesculin, starch, tyrosine, and casein hydrolysis and reduction of nitrates.

STAPHYtest 24 positive for sucrose, trehalose, maltose, and mannose acidification and acetoin production. Variable for phosphatase, N-acetyl-β-D-glucosamine and galactose fermentation. Negative for urea hydrolysis, arginine dihydrolase, ornithine decarboxylase, ß-galactosidase, ß-glucuronidase, ß-glucosidase, aesculin hydrolysis, mannitol, xylose, lactose, sorbitol, ribose, fructose, cellobiose, arabinose, raffinose, and xylitol acidification.

API ZYM positive for lipase (C14), leucine arylamidase, valine arylamidase, naphthol-AS-Bl-phosphohydrolase, and α-glucosidase. Weakly positive for esterase lipase (C8) and cystine arylamidase. Negative for alkaline phosphatase, esterase (C4), trypsin, α-chymotrypsin, acid phosphatase, α-galactosidase, β-galactosidase, β-glucuronidase, β-glucosidase, N-acetyl-β-glucosaminidase, α-mannosidase, and α-fucosidase.

The type strain, P7182^T^ (= CCM 9418^T^ = LMG 33814^T^), was isolated from Adélie penguin (*Pygoscelis adeliae*) oral cavity at James Ross Island in the Antarctic Peninsula region. The genomic G+C content of the type strain is 47.5 mol%. The GenBank/EMBL/DDBJ accession number for the 16S rRNA sequence of the type strain is PV222028, and the whole-genome sequence accession number is JBLXXX000000000.

### *Rothia antarctica* sp. nov.

ant.arc'ti.ca. L. fem. adj. *antarctica*, southern, pertaining to the Antarctic, where the type strain was isolated.

Cells are Gram-variable, non-spore forming, non-motile, coccoid, 0.5–1.3 μm in diameter, varying in size, occurring in clusters. Grows aerobically. Forms raised, creamy white, smooth colonies with entire margin when cultivated on TSA at 30°C for 48 h. Grows on TSA and blood agar. Grows at 25°C–37°C. Tolerates up to 7% NaCl (w/v). The major cellular fatty acid is C_15:0_  *anteiso*.

Positive for catalase production and gelatine hydrolysis. Weakly positive for DNA hydrolysis. Negative for production of acetoin, pyrrolidonyl arylamidase, β-galactosidase (ONPG test), lecithinase (egg-yolk reaction), and urease. Negative for Tween 80, arginine, aesculin, starch, tyrosine, and casein hydrolysis and reduction of nitrates.

STAPHYtest 24 positive for sucrose, maltose, and mannose acidification. Variable for galactose and fructose fermentation and acetoin production. Negative for urea hydrolysis, arginine dihydrolase, ornithine decarboxylase, ß-galactosidase, ß-glucuronidase, ß-glucosidase, phosphatase, aesculin hydrolysis, N-acetyl-β-D-glucosamine, trehalose, mannitol, xylose, lactose, sorbitol, ribose, cellobiose, arabinose, raffinose, and xylitol acidification.

API ZYM positive for alkaline phosphatase, lipase (C14), leucine arylamidase, valine arylamidase, naphthol-AS-Bl-phosphohydrolase, and α-glucosidase. Variable for esterase lipase (C8). Negative for esterase (C4), cystine arylamidase, trypsin, α-chymotrypsin, acid phosphatase, α-galactosidase, β-galactosidase, β-glucuronidase, β-glucosidase, N-acetyl-β-glucosaminidase, α-mannosidase, and α-fucosidase.

The type strain, P7162^T^ (= CCM 9419^T^ = LMG 33813^T^), was isolated from Gentoo penguin (*Pygoscelis papua*) oral cavity at James Ross Island in the Antarctic Peninsula region. The genomic G+C content of the type strain is 47.5 mol%. The GenBank/EMBL/DDBJ accession number for the 16S rRNA sequence of the type strain is PV222026, and the whole-genome sequence accession number is JBLXXY000000000.

## Supplementary Material

fiag056_Supplemental_File

## Data Availability

The 16S rRNA gene sequences have been stored in the NCBI Nucleotide database and are available under the following accession numbers: PV222020.1 (P4278), PV222021.1 (P5758 (= CCM 9416)), PV222022.1 (P5764), PV222023.1 (P5766), PV222024.1 (P5771^T^ = CCM 9417^T^), PV222025.1 (P6271), PV222028.1 (P7182^T^ = CCM 9418^T^), PV222029.1 (P7208), PV222026.2 (P7162^T^ = CCM 9419^T^), PV222027.2 (P7181), PV222019.2 (P13129). The draft whole-genome sequences have been stored in the NCBI Nucleotide database and are available under the IDs JBLXXU000000000 (P4278), JBLXXR000000000 (P5758 (= CCM 9416)), JBLXXQ000000000 (P5764), JBLXXS000000000 (P5766), JBLXXT000000000 (P5771^T^ (= CCM 9417^T^)), JBLXXV000000000 (P6271), JBLXXX000000000 (P7182^T^ (= CCM 9418^T^)), JBLXXW000000000 (P7208), JBLXXY000000000 (P7162^T^ (= CCM 9419^T^)), JBLXXZ000000000 (P7181), and JBLXYA000000000 (P13129). Sequencing reads have been deposited in the NCBI Sequence Read Archive (SRA) under the accession numbers SRR33542411 (P4278), SRR33542404 (P5758 (= CCM 9416)), SRR33542405 (P5764), SRR33542413 (P5766), SRR33542412 (P5771^T^ (= CCM 9417^T^)), SRR33542410 (P6271), SRR33542408 (P7182^T^ (= CCM 9418^T^)), SRR33542409 (P7208), SRR33542407 (P7162^T^ (= CCM 9419^T^)), SRR33542406 (P7181), and SRR33542403 (P13129).
