# Supplementary material for: Three novel Rothia species associated with Antarctic birds harbour novel biosynthetic gene clusters
Source: FEMS Microbiol Ecol. 2026 May 29;102(7):fiag056. doi: 10.1093/femsec/fiag056 (PMC13347742; doi:10.1093/femsec/fiag056)
Supplement: fiag056_Supplemental_File [file fiag056_supplemental_file.docx]

# Supplementary data

# Three novel *Rothia* species associated with Antarctic birds harbour novel biosynthetic gene clusters

Vendula Koublová^1, 2^, Jana Musilova^3^, Karel Sedlar^3^, Peter Spacek^4,5^, Jitka Vives^1^, Eva Staňková^1^, Ondrej Šedo^6^, Stanislava Kralova^4,5^, Luděk Sehnal^1^, Ivo Sedláček^1^, Pavel Švec^1^

^1^Department of Experimental Biology, Czech Collection of Microorganisms, Faculty of Science, Masaryk University, Kamenice 5, 625 00 Brno, Czech Republic

^2^Czech Academy of Sciences, Global Change Research Institute, Bělidla 986/4a, 603 00 Brno, Czech Republic

^3^Department of Biomedical Engineering, Faculty of Electrical Engineering and Communication, Brno University of Technology, Technická 12, 616 00 Brno, Czech Republic

^4^Department of Molecular Pharmacy, Faculty of Pharmacy, Masaryk University, Palackého třída 1946/1, 612 00 Brno, Czech Republic

^5^Department of Chemistry and Biochemistry, Faculty of AgriSciences, Mendel University in Brno, Zemědělská 1665/1, 613 00 Brno, Czech Republic

^6^Central European Institute of Technology, Masaryk University, Kamenice 5, 625 00, Brno, Czech Republic

*Corresponding author: Vendula Koublová, e-mail: koublova@mail.muni.cz


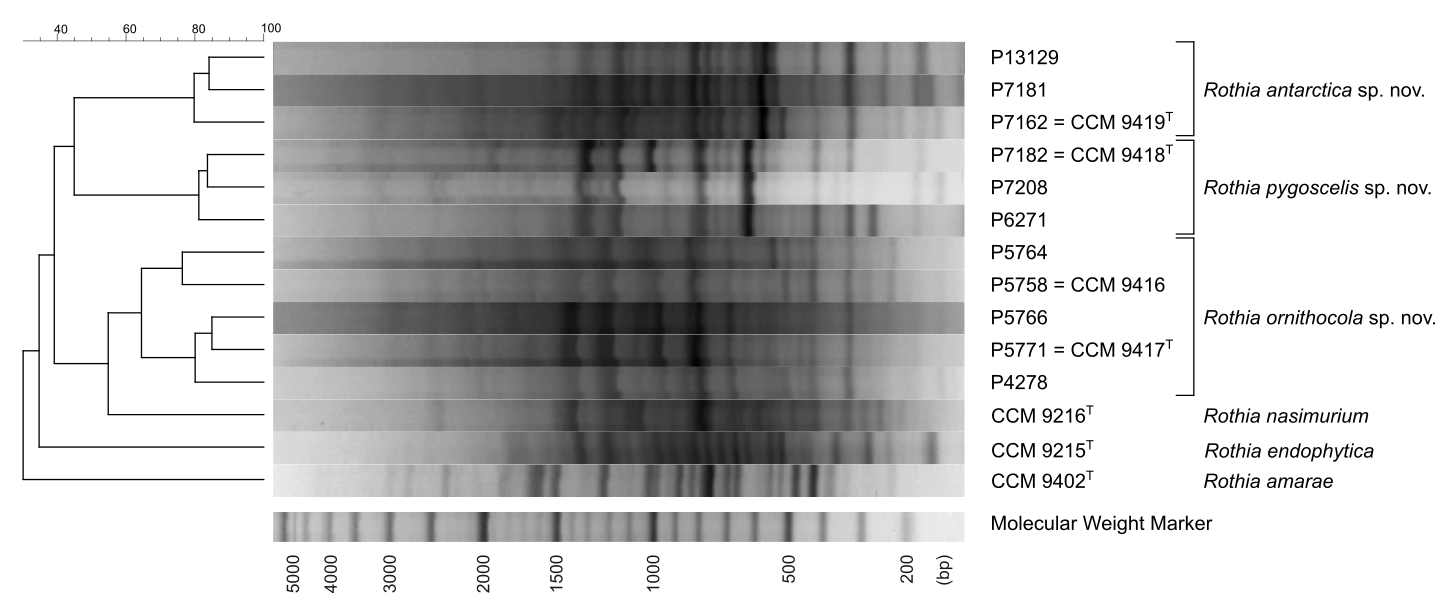


**Supplementary Figure S1.** Dendrogram based on cluster analysis of rep-PCR fingerprints obtained with (GTG)_5_ primer from the studied *Rothia* strains and reference type strains.


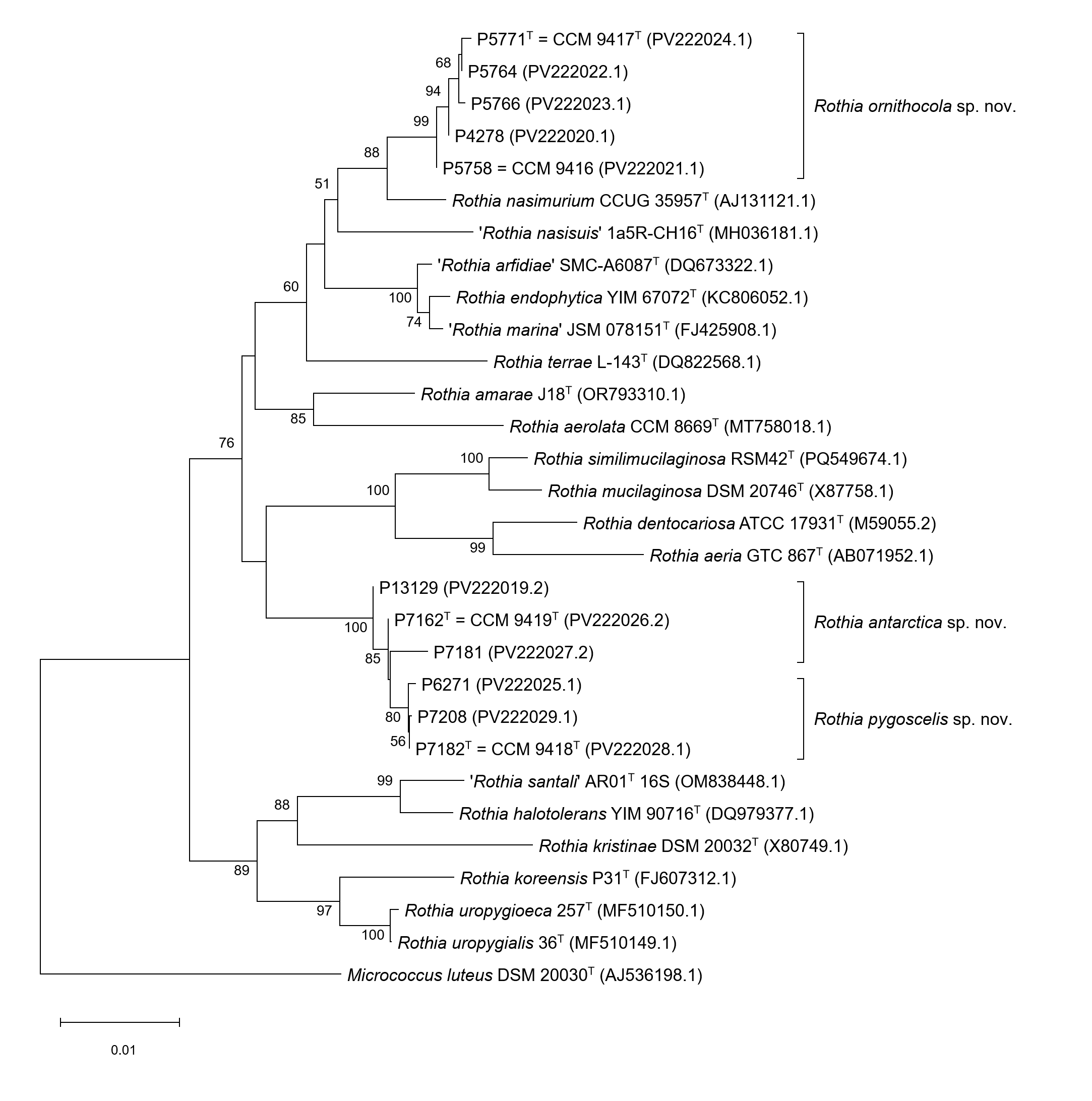


**Supplementary Figure S2.** Minimum Evolution phylogenetic tree based on 16S rRNA sequences showing the position of the studied strains within the genus *Rothia*. Numbers at nodes represent bootstrap values of 50 % and above. The final dataset included 1546 positions. *Micrococcus luteus* DSM 20030^T^ was used as an outgroup. Bar, 0.01 substitutions per nucleotide position.


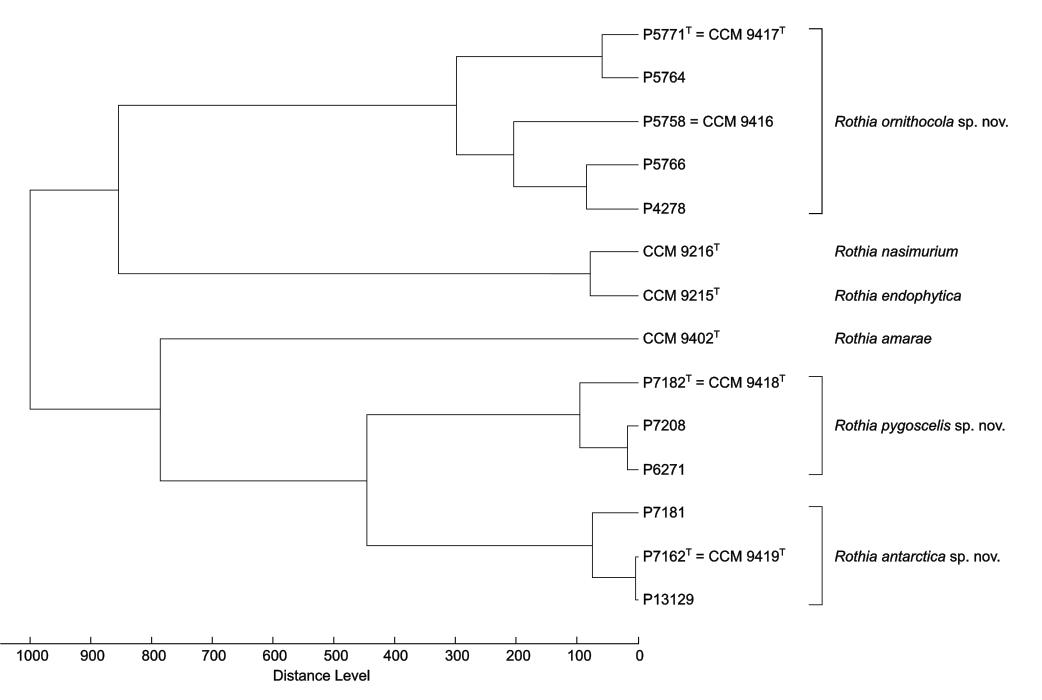


**Supplementary Figure S3.** Dendrogram based on cluster analysis of MALDI-TOF mass spectra of the studied *Rothia* strains and three reference strains.


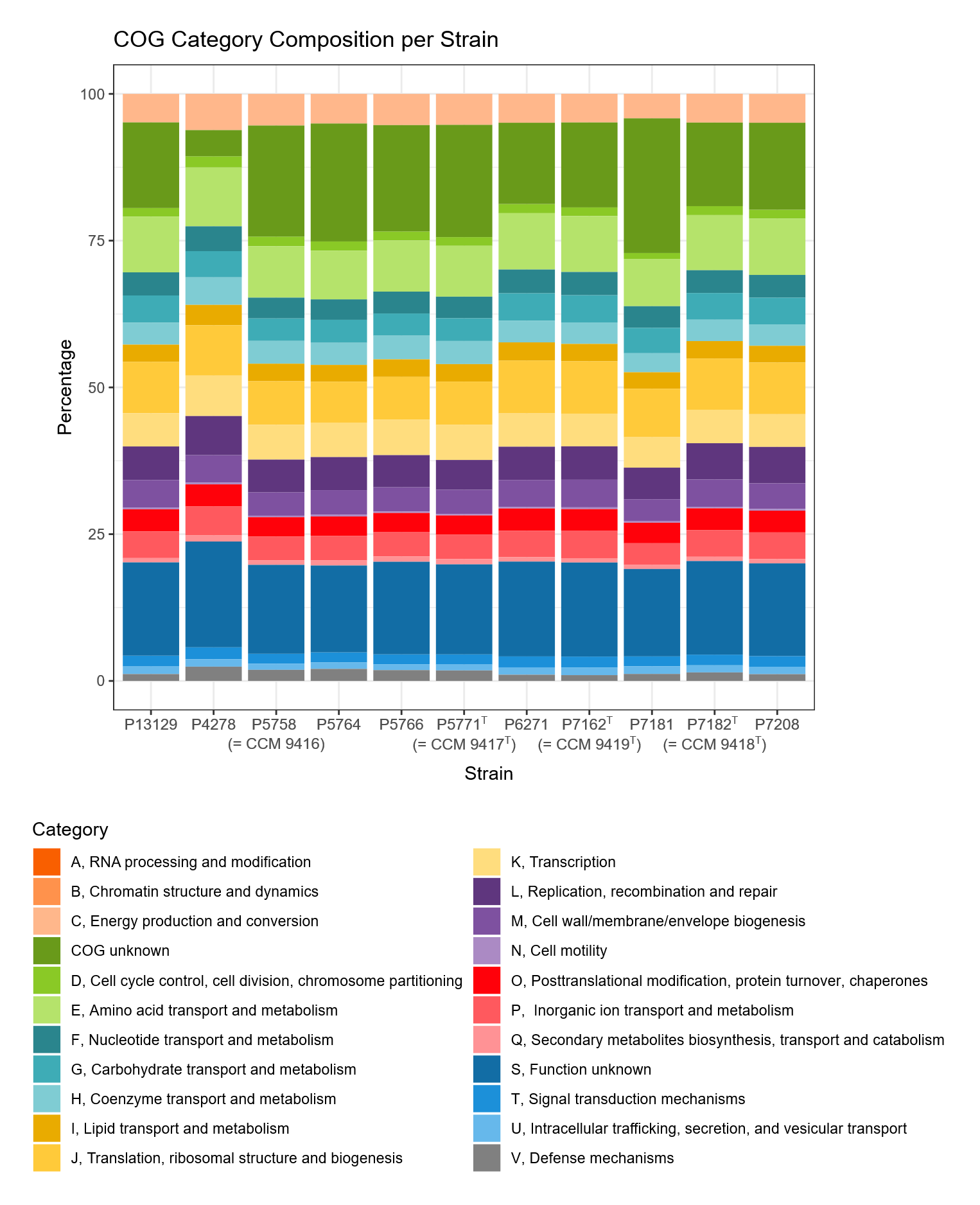


**Supplementary Figure S4.** Relative abundances of genes in particular clusters of orthologous groups (COGs).


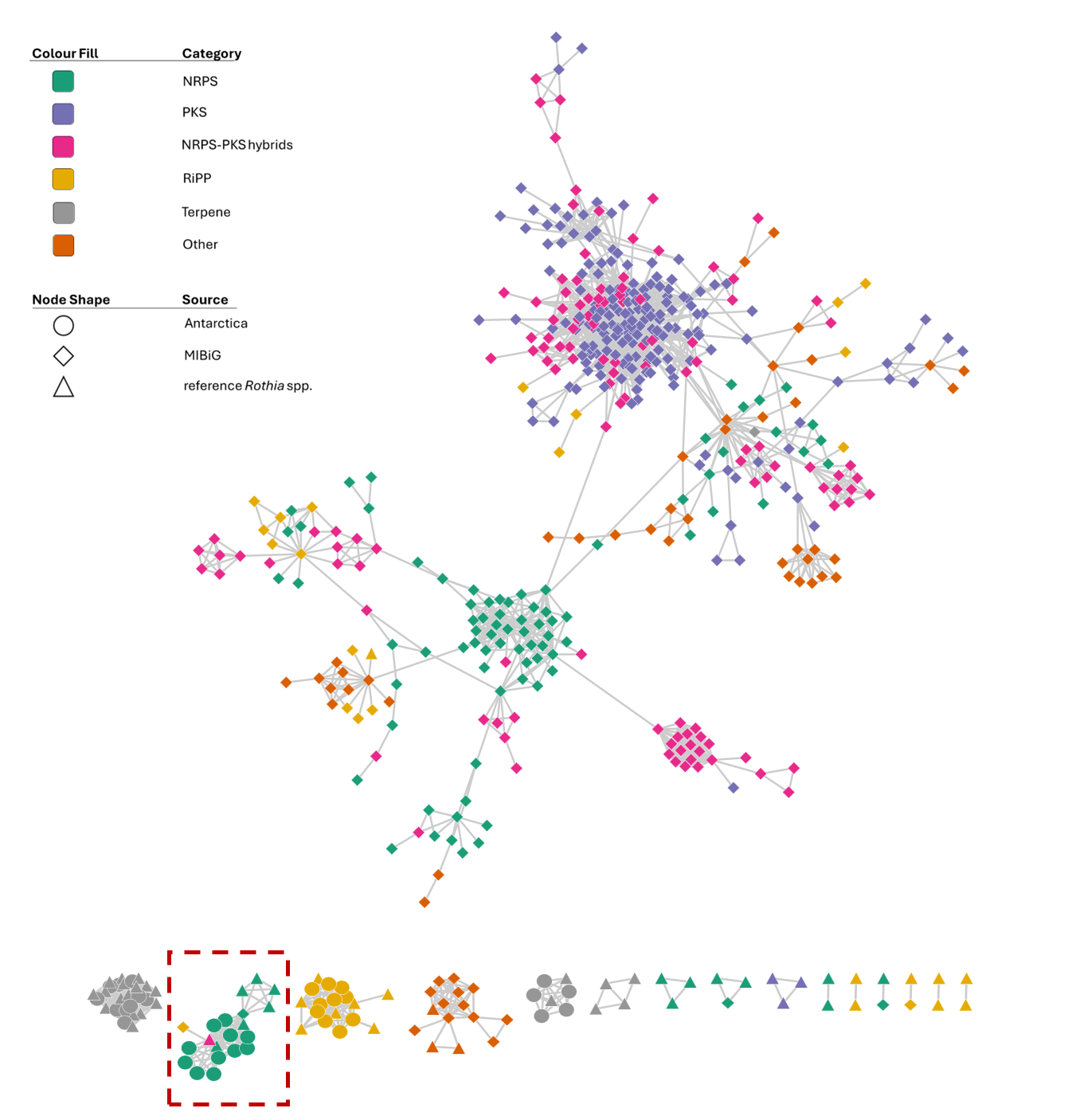


**Supplementary Figure S5.** BiG-SCAPE (v2.0.0) sequence similarity network (SSN) generated at a cut-off of c = 0.5 for *Rothia* spp. and visualized in Cytoscape (v3.10.3), including MIBiG v3.1 reference BGCs. The enterobactin-associated GCF is highlighted by a boxed region.

**Supplementary Table S1.** List of *Rothia* strains presented in this study.

| Strain | Source | Location | Coordinates | Year | 16S rRNA | Genome |
| --- | --- | --- | --- | --- | --- | --- |
| *Rothia ornithocola* sp. nov. | |  |  |  |  |  |
| P4278 | Feathers and bird faeces | James Ross Island | 63°46'42" S 57°46'54" W | 2011 | PV222020.1 | JBLXXU000000000 |
| P5758 = CCM 9416 | Unknown bird faeces | Seymour Island | 64°17'22" S 56°41'13" W | 2014 | PV222021.1 | JBLXXR000000000 |
| P5764 | Kelp gull faeces | Seymour Island | 64°17'22" S 56°41'13" W | 2014 | PV222022.1 | JBLXXQ000000000 |
| P5766 | Kelp gull faeces | Seymour Island | 64°17'22" S 56°41'13" W | 2014 | PV222023.1 | JBLXXS000000000 |
| P5771^T^ = CCM 9417^T^ | Skua faeces | James Ross Island | 63°47'58" S 57°48'42" W | 2014 | PV222024.1 | JBLXXT000000000 |
| *Rothia pygoscelis* sp. nov. | |  |  |  |  |  |
| P6271 | Adélie penguin, oral cavity | Seymour Island | 64°17'22" S 56°41'13" W | 2014 | PV222025.1 | JBLXXV000000000 |
| P7182^T^ = CCM 9418^T^ | Adélie penguin, oral cavity | James Ross Island | 63°48'01" S 57°53'02" W | 2016 | PV222028.1 | JBLXXX000000000 |
| P7208 | Adélie penguin, oral cavity | James Ross Island | 63°48'01" S 57°53'02" W | 2016 | PV222029.1 | JBLXXW000000000 |
| *Rothia* *antarctica* sp. nov. | |  |  |  |  |  |
| P7162^T^ = CCM 9419^T^ | Gentoo penguin, oral cavity | James Ross Island | 63°48'01" S 57°53'02" W | 2016 | PV222026.2 | JBLXXY000000000 |
| P7181 | Adélie penguin, oral cavity | James Ross Island | 63°48'01" S 57°53'02" W | 2016 | PV222027.2 | JBLXXZ000000000 |
| P13129 | Gentoo penguin, oral cavity | James Ross Island | 63°48'01" S 57°53'02" W | 2021 | PV222019.2 | JBLXYA000000000 |

**Supplementary Table S2.** Reference MALDI-TOF MS peak lists for novel *Rothia* spp.

| ***Rothia ornithocola* sp. nov.**  **CCM 9417^T^** | | ***Rothia pygoscelea* sp. nov.**  **CCM 9418^T^** | | ***Rothia antarctica* sp. nov.**  **CCM 9419^T^** | |
| --- | --- | --- | --- | --- | --- |
| **m/z** | **Relative intensity (%)** | **m/z** | **Relative intensity (%)** | **m/z** | **Relative intensity (%)** |
| 2149 | 6.48 | 2152 | 1.58 | 3303 | 22.58 |
| 2572 | 5.51 | 2246 | 0.80 | 3322 | 8.24 |
| 2648 | 5.03 | 2308 | 1.95 | 3341 | 3.17 |
| 3244 | 11.83 | 3277 | 4.36 | 3354 | 1.49 |
| 3732 | 10.91 | 3289 | 14.83 | 3703 | 1.62 |
| 3760 | 8.86 | 3699 | 2.45 | 3752 | 2.51 |
| 3969 | 21.99 | 3752 | 1.30 | 4305 | 5.17 |
| 3996 | 5.05 | 4274 | 2.36 | 4493 | 4.88 |
| 4125 | 6.36 | 4291 | 6.11 | 4937 | 4.83 |
| 4270 | 28.11 | 4306 | 18.09 | 4954 | 1.91 |
| 4298 | 74.37 | 4319 | 5.63 | 5864 | 2.07 |
| 4314 | 27.34 | 4341 | 3.72 | 6377 | 1.62 |
| 4451 | 9.70 | 4463 | 2.82 | 6518 | 2.92 |
| 4695 | 12.54 | 4479 | 3.01 | 6605 | 100.00 |
| 4879 | 7.32 | 4493 | 10.76 | 6628 | 41.32 |
| 4895 | 26.81 | 4530 | 3.72 | 6643 | 44.10 |
| 4906 | 12.64 | 4936 | 3.39 | 6664 | 19.00 |
| 5136 | 7.47 | 4952 | 1.11 | 6680 | 13.69 |
| 5155 | 11.88 | 5835 | 4.27 | 6740 | 4.50 |
| 5957 | 3.87 | 5964 | 1.07 | 7404 | 2.08 |
| 6347 | 11.48 | 6086 | 1.23 | 7501 | 3.50 |
| 6487 | 37.66 | 6367 | 0.77 | 7529 | 1.37 |
| 6773 | 8.27 | 6490 | 3.17 | 7539 | 1.21 |
| 7461 | 28.79 | 6577 | 100.00 | 7549 | 0.92 |
| 7518 | 22.37 | 6600 | 45.37 | 7563 | 0.66 |
| 7864 | 16.91 | 6614 | 48.80 | 7573 | 0.62 |
| 7936 | 100.00 | 6710 | 6.83 | 8582 | 0.63 |
| 7957 | 45.75 | 6724 | 6.68 | 8923 | 3.07 |
| 8244 | 10.52 | 6786 | 2.97 | 9872 | 4.23 |
| 8452 | 9.90 | 7403 | 6.34 | 9902 | 1.44 |
| 8537 | 40.53 | 7501 | 3.49 |  |  |
| 8580 | 38.91 | 8565 | 2.12 |  |  |
| 8901 | 7.92 | 8583 | 3.09 |  |  |
| 9389 | 16.24 | 8922 | 2.17 |  |  |
| 9705 | 4.93 | 9870 | 5.11 |  |  |
| 9787 | 49.03 | 9902 | 1.77 |  |  |
| 10309 | 7.10 | 11929 | 0.33 |  |  |
| 11911 | 1.64 |  |  |  |  |
| 12691 | 3.52 |  |  |  |  |

**Supplementary Table S3.** Reference strains used for the bioinformatic analyses.

| **Species** | **Strain designation** | **Assembly accession number** | **Isolation source** | **Country of origin** |
| --- | --- | --- | --- | --- |
| *Rothia aeria* | FDAARGOS_1137^T^ | GCF_016726365.1 | air in space station Mir | space (Earth orbit) |
| *Rothia aerolata* | CCM 8669^T^ | GCF_014635585.1 | exhaust air of a pig barn | Germany |
| *Rothia amarae* | JCM 11375^T^ | GCF_042677725.1 | sludge of a foul water sewer | China |
| *´Candidatus* Rothia avicola*´* | ChiHjej12B11-9195 | GCA_019115985.1 | adult hen faeces (*Gallus gallus*) | United Kingdom |
| *´Candidatus* Rothia avistercoris*´* | ChiHjej10B9-4811 | GCA_019120655.1 | adult hen faeces (*Gallus gallus*) | United Kingdom |
| *Rothia dentocariosa* | ATCC 17931^T^ | GCF_000164695.2 | human caries lesion | NA |
| *Rothia endophytica* | JCM 18541^T^ | GCF_039543885.1 | healthy roots of *Dysophylla stellata* | China |
| *Rothia halotolerans* | JCM 31975^T^ | GCF_004136635.1 | saline soil sample | China |
| *Rothia koreensis* | JCM 15915^T^ | GCF_004136575.1 | Korean traditional fermented seafood (jeotgal) | South Korea |
| *Rothia kristinae* | FDAARGOS_864^T^ | GCF_016028855.1 | healthy human skin | United States of America |
| *Rothia mucilaginosa* | ATCC 25296^T^ | GCF_000175615.1 | human throat, patient with sinusitis | Norway |
| *Rothia nasimurium* | CCUG 35957^T^ | GCF_042660305.1 | nose of a healthy mouse | Sweden |
| *´Rothia nasisuis´* | 69RC1 | GCF_928381405.1 | tonsil of healthy piglet (*Sus scrofa*) | Spain |
| *´Rothia santali´* | AR01^T^ | GCF_024199905.1 | sandalwood seedling (*Santalum album*) | India |
| *Rothia similimucilaginosa* | RSM42^T^ | GCF_029850985.2 | nasal lavage samples collected from children | United States of America |
| *Rothia terrae* | LMG 23708^T^ | GCF_012396615.1 | wasteland soil | Taiwan |
| *Rothia uropygialis* | 36^T^ | GCF_004137765.1 | oil gland of a live woodpecker (*Dendrocopos major*) | Germany |
| *Rothia uropygioeca* | 257^T^ | GCF_004136585.1 | oil gland of a live woodpecker (*Dendrocopos major*) | Germany |

The information on isolation source and country was obtained either from the GenBank database or the original species description.

**Supplementary Table S4.** Genomic features of the studied *Rothia* strains.

|  | ***R. ornithocola* sp. nov.** | | | | | ***R. pygoscelis* sp. nov.** | | | ***R. antarctica* sp. nov.** | | |
| --- | --- | --- | --- | --- | --- | --- | --- | --- | --- | --- | --- |
| **Feature** | **P4278** | **P5758 = CCM 9416** | **P5764** | **P5766** | **P5771 ^T^ = CCM 9417 ^T^** | **P6271** | **P7182^T^ = CCM 9418^T^** | **P7208** | **P7162 ^T^  = CCM 9419 ^T^** | **P7181** | **P13129** |
| Length (bp) | 2 354 857 | 2 285 120 | 2 407 818 | 2 285 078 | 2 293 321 | 1 850 320 | 1 881 589 | 1 893 386 | 1 853 352 | 1 827 380 | 1 872 372 |
| Contigs | 10 | 40 | 19 | 31 | 17 | 16 | 7 | 14 | 12 | 25 | 14 |
| N50 | 653 203 | 80 936 | 296 458 | 133 134 | 409 210 | 146 513 | 402 954 | 285 913 | 303 969 | 121 216 | 187 763 |
| L50 | 2 | 8 | 3 | 7 | 2 | 3 | 2 | 2 | 3 | 6 | 4 |
| Completeness | 100 | 99.92 | 100 | 100 | 100 | 99.99 | 100 | 100 | 99.99 | 99.81 | 99.99 |
| Contamination | 0.03 | 0 | 0.02 | 0.02 | 0.02 | 0 | 0 | 0 | 0 | 0 | 0 |
| GC content (%) | 56.1 | 56.2 | 56.1 | 56.2 | 56.3 | 47.4 | 47.4 | 47.4 | 47.5 | 47.5 | 47.5 |
| ORFs | 2104 | 2071 | 2174 | 2076 | 2074 | 1667 | 1714 | 1717 | 1674 | 1662 | 1682 |
| CDSs | 2040 | 2008 | 2096 | 2010 | 2009 | 1604 | 1650 | 1648 | 1617 | 1600 | 1625 |
| Pseudogenes | 6 | 13 | 15 | 9 | 10 | 6 | 7 | 12 | 2 | 7 | 3 |
| rRNAs (5S, 16S, 23S) | 2, 1, 1 | 0, 1, 1 | 2, 2, 4 | 2, 1, 1 | 0, 1, 1 | 4, 1, 1 | 4, 1, 1 | 4, 1, 1 | 3, 1, 1 | 4, 1, 1 | 4, 1, 1 |
| tRNAs | 51 | 45 | 52 | 50 | 50 | 48 | 48 | 48 | 47 | 46 | 46 |
| ncRNAs | 3 | 3 | 3 | 3 | 3 | 3 | 3 | 3 | 3 | 3 | 2 |

**Supplementary Table S5.** Inhibition zones (mm) of the tested strains and the reference type strains *R. nasimurium* CCM 9216^T^, *R. endophytica* CCM 9215^T^ and *R. amarae* CCM 9402^T^. Breakpoint values are shown in the top part of the table.

| **Strain** | R < 18 | R < 25 | R < 25 | R < 20 | R < 24 | R < 25 | R < 22 | R < 21 | R < 25 | R < 21 | R < 12 | R < 30 | R < 23 | R < 24 | R < 17 |
| --- | --- | --- | --- | --- | --- | --- | --- | --- | --- | --- | --- | --- | --- | --- | --- |
|  | S ≥ 18 | S ≥ 25 | S ≥ 50 | S ≥ 20 | S ≥ 24 | S ≥ 25 | S ≥ 22 | S ≥ 21 | S ≥ 25 | S ≥ 50 | S ≥ 50 | S ≥ 30 | S ≥ 23 | S ≥ 24 | S ≥ 17 |
|  | **AMP** | **FEP** | **CIP** | **DA** | **E** | **MXF** | **CN** | **C** | **LNZ** | **IPM** | **P** | **RA** | **SXT** | **TE** | **VA** |
|  | 10 µg | 30 µg | 5 µg | 2 µg | 15 µg | 10 µg | 10 µg | 30 µg | 10 µg | 1 µg | 1 IU | 5 µg | 25 µg | 30 µg | 15 µg |
| ***R. ornithocola* sp. nov.** |  |  |  |  |  |  |  |  |  |  |  |  |  |  |  |
| P4278 | **R** | **24** | *36* | 40 | 48 | 48 | 28 | 50 | 44 | **R** | **R** | 44 | 46 | 50 | 24 |
| P5758 = CCM 9416 | **R** | 26 | *48* | 42 | 44 | 50 | 30 | 60 | 50 | **R** | **R** | 60 | 42 | 60 | 26 |
| P5764 | **R** | **20** | *40* | 42 | 52 | 50 | 26 | 52 | 44 | **R** | **10** | 50 | 46 | 44 | 26 |
| P5766 | **11** | **20** | *32* | 40 | 48 | 40 | 24 | 50 | 44 | **R** | **R** | 40 | 40 | 44 | 22 |
| P5771^T^ = CCM 9417^T^ | **R** | 28 | *40* | 36 | 48 | 30 | 24 | 52 | 40 | **R** | **R** | 44 | 38 | 44 | 24 |
| ***R. pygoscelis* sp. nov.** |  |  |  |  |  |  |  |  |  |  |  |  |  |  |  |
| P6271 | **R** | **R** | S | S | S | S | S | S | S | S | **R** | S | 45 | S | S |
| P7182^T^ = CCM 9418^T^ | **R** | 36 | *44* | 40 | 38 | 70 | 52 | 46 | 34 | **R** | **R** | 40 | 40 | 32 | 24 |
| P7208 | **R** | **24** | S | 44 | S | S | S | S | S | **R** | **R** | S | 36 | S | 36 |
| ***R. antarctica* sp. nov.** |  |  |  |  |  |  |  |  |  |  |  |  |  |  |  |
| P7162^T^ = CCM 9419^T^ | 24 | 40 | S | S | S | S | S | S | S | **R** | **R** | S | 40 | S | 36 |
| P7181 | S | S | S | S | S | S | S | S | S | S | **R** | S | S | S | S |
| P13129 | **16** | 42 | *40* | 44 | S | S | S | S | S | **R** | **R** | S | 42 | S | 34 |
| **CCM 9215^T^** | **R** | 25 | *32* | **R** | **R** | 36 | 28 | **10** | 38 | **R** | **R** | **R** | 30 | 40 | 24 |
| **CCM 9216^T^** | **16** | 32 | *35* | **R** | **R** | 40 | 30 | **R** | 40 | **R** | **R** | **R** | 40 | 30 | 26 |
| **CCM 9402^T^** | **R** | **R** | *36* | **19** | 60 | 44 | 30 | 50 | 44 | **R** | **R** | 60 | 54 | 42 | 27 |

AMP, ampicillin; FEP, cefepime; CIP, ciprofloxacin; DA, clindamycin; E, erythromycin; MXF, moxifloxacin; CN, gentamicin; C, chloramphenicol; LNZ, linezolid; IPM, imipenem; P, penicillin G; RA, rifampicin; SXT, trimethoprim/sulfamethoxazole; TE, tetracycline; VA, vancomycin

R, no inhibition zone, growth up to the edges of cellulose disc; S, zone too large to be measured

Resistant in bold, Intermediate in italics, susceptible in regular

**Supplementary Table S6.** Cellular fatty acid composition (%) of the studied Antarctic *Rothia* strains and the type reference strains *R. nasimurium* CCM 9216^T^, *R. endophytica* CCM 9215^T^ and *R. amarae* CCM 9402^T^. Major cellular fatty acids are in bold.

|  | ***R. ornithocola* sp. nov.** | | | | | ***R. pygoscelis* sp. nov.** | | | ***R. antarctica* sp. nov.** | | | **Reference strains** | | |
| --- | --- | --- | --- | --- | --- | --- | --- | --- | --- | --- | --- | --- | --- | --- |
| **Fatty acid** | **P5764** | **P5758 = CCM 9416** | **P5766** | **P5771^T^ = CCM 9417^T^** | **P4278** | **P6271** | **P7208** | **P7182^T^ = CCM 9418^T^** | **P7162^T^ = CCM 9419^T^** | **P7181** | **P13129** | **CCM 9216^T^** | **CCM 9402^T^** | **CCM 9215^T^** |
| C_13:0_ iso | TR | ND | ND | ND | ND | 1.1 | 2.1 | 1.1 | 1.1 | 1.0 | 1.6 | TR | ND | TR |
| C_13:0_ anteiso | 6.8 | 5.4 | 8.2 | 4.7 | 5.5 | 7.4 | 14.0 | 7.3 | 7.3 | 6.4 | 9.0 | 8.8 | TR | 3.1 |
| C_14:0_ iso | 2.7 | 1.9 | 2.0 | 2.8 | 3.0 | 6.6 | 8.8 | 8.7 | 8.6 | 7.4 | 8.6 | 6.9 | 1.2 | 2.3 |
| C_14:0_ | TR | TR | TR | TR | TR | 1.5 | 1.2 | 1.0 | 1.7 | 1.6 | 1.3 | 5.3 | TR | 1.1 |
| C_15:0_ iso | 1.8 | 2.1 | 1.9 | 2.0 | 1.7 | 6.4 | 5.9 | 6.7 | 7.4 | 8.6 | 8.9 | 2.7 | 6.8 | 3.3 |
| **C_15:0_ anteiso** | **82.8** | **83.9** | **82.5** | **84.6** | **83.2** | **71.8** | **64.7** | **70.8** | **67.7** | **69.9** | **66.5** | **59.2** | **71.3** | **81.6** |
| C_16:0_ iso | 2.8 | 3.0 | 2.3 | 3.5 | 3.3 | 2.2 | 1.2 | 2.1 | 3.1 | 2.7 | 2.1 | 10.0 | 6.4 | 3.8 |
| C_16:0_ | TR | TR | TR | TR | TR | 1.0 | TR | TR | TR | TR | TR | 2.7 | 2.6 | TR |
| C_17:0_ anteiso | 1.3 | 2.3 | 1.6 | 1.4 | 1.6 | 1.7 | TR | TR | 1.2 | 1.1 | TR | 2.4 | 10.1 | 3.4 |

TR – trace amounts < 1 %; ND - not detected

**Supplementary Table S7.** Clusters of orthologous groups (COGs) identified by eggNOG-mapper v2.1.12 in studied Antarctic *Rothia* strains.

| **COG class** | ***Rothia ornithocola* sp. nov.** | | | | | | | | | | ***Rothia pygoscelis* sp. nov.** | | | | | | ***Rothia antarctica* sp. nov.** | | | | | |
| --- | --- | --- | --- | --- | --- | --- | --- | --- | --- | --- | --- | --- | --- | --- | --- | --- | --- | --- | --- | --- | --- | --- |
|  | **P4278** | | **P5758 = CCM 9416** | | **P5764** | | **P5766** | | **P5771^T^ = CCM 9417^T^** | | **P6271** | | **P7182^T^ = CCM 9418^T^** | | **P7208** | | **P7162^T^ = CCM 9419^T^** | | **P7181** | | **P13129** | |
|  | **Count** | **%** | **Count** | **%** | **Count** | **%** | **Count** | **%** | **Count** | **%** | **Count** | **%** | **Count** | **%** | **Count** | **%** | **Count** | **%** | **Count** | **%** | **Count** | **%** |
| A, RNA processing and modification | 0 | 0.00 | 0 | 0.00 | 0 | 0.00 | 0 | 0.00 | 0 | 0.00 | 0 | 0.00 | 0 | 0.00 | 0 | 0.00 | 0 | 0.00 | 0 | 0.00 | 0 | 0.00 |
| B, Chromatin structure and dynamics | 1 | 0.06 | 1 | 0.05 | 1 | 0.05 | 1 | 0.05 | 1 | 0.05 | 0 | 0.00 | 0 | 0.00 | 0 | 0.00 | 0 | 0.00 | 0 | 0.00 | 0 | 0.00 |
| C, Energy production and conversion | 105 | 6.08 | 107 | 5.33 | 105 | 5.01 | 106 | 5.27 | 105 | 5.23 | 79 | 4.93 | 81 | 4.91 | 81 | 4.92 | 79 | 4.89 | 67 | 4.19 | 79 | 4.86 |
| D, Cell cycle control, cell division, chromosome partitioning | 33 | 1.91 | 33 | 1.64 | 33 | 1.57 | 31 | 1.54 | 30 | 1.49 | 25 | 1.56 | 25 | 1.52 | 25 | 1.52 | 24 | 1.48 | 16 | 1.00 | 24 | 1.48 |
| E, Amino acid transport and metabolism | 173 | 10.02 | 176 | 8.76 | 174 | 8.30 | 175 | 8.71 | 174 | 8.66 | 154 | 9.60 | 155 | 9.39 | 158 | 9.59 | 154 | 9.52 | 129 | 8.06 | 154 | 9.48 |
| F, Nucleotide transport and metabolism | 73 | 4.23 | 71 | 3.54 | 73 | 3.48 | 75 | 3.73 | 74 | 3.68 | 65 | 4.05 | 64 | 3.88 | 64 | 3.88 | 64 | 3.96 | 59 | 3.69 | 64 | 3.94 |
| G, Carbohydrate transport and metabolism | 77 | 4.46 | 77 | 3.83 | 82 | 3.91 | 76 | 3.78 | 78 | 3.88 | 75 | 4.68 | 75 | 4.55 | 75 | 4.55 | 75 | 4.64 | 69 | 4.31 | 75 | 4.62 |
| H, Coenzyme transport and metabolism | 81 | 4.69 | 78 | 3.88 | 79 | 3.77 | 81 | 4.03 | 79 | 3.93 | 59 | 3.68 | 60 | 3.64 | 60 | 3.64 | 59 | 3.65 | 52 | 3.25 | 61 | 3.75 |
| I, Lipid transport and metabolism | 60 | 3.48 | 60 | 2.99 | 60 | 2.86 | 60 | 2.99 | 60 | 2.99 | 50 | 3.12 | 49 | 2.97 | 47 | 2.85 | 48 | 2.97 | 45 | 2.81 | 48 | 2.95 |
| J, Translation, ribosomal structure and biogenesis | 148 | 8.57 | 149 | 7.42 | 146 | 6.97 | 146 | 7.26 | 147 | 7.32 | 144 | 8.98 | 144 | 8.73 | 145 | 8.80 | 144 | 8.91 | 132 | 8.25 | 142 | 8.74 |
| K, Transcription | 119 | 6.89 | 119 | 5.93 | 123 | 5.87 | 122 | 6.07 | 121 | 6.02 | 91 | 5.67 | 94 | 5.70 | 92 | 5.58 | 90 | 5.57 | 83 | 5.19 | 92 | 5.66 |
| L, Replication, recombination and repair | 115 | 6.66 | 112 | 5.58 | 118 | 5.63 | 110 | 5.47 | 102 | 5.08 | 91 | 5.67 | 102 | 6.18 | 102 | 6.19 | 92 | 5.69 | 86 | 5.38 | 93 | 5.72 |
| M, Cell wall/membrane/envelope biogenesis | 81 | 4.69 | 80 | 3.98 | 88 | 4.20 | 83 | 4.13 | 83 | 4.13 | 74 | 4.61 | 77 | 4.67 | 72 | 4.37 | 76 | 4.70 | 59 | 3.69 | 76 | 4.68 |
| N, Cell motility | 5 | 0.29 | 5 | 0.25 | 6 | 0.29 | 5 | 0.25 | 5 | 0.25 | 4 | 0.25 | 4 | 0.24 | 5 | 0.30 | 5 | 0.31 | 5 | 0.31 | 5 | 0.31 |
| O, Posttranslational modification, protein turnover, chaperones | 65 | 3.77 | 66 | 3.29 | 69 | 3.29 | 66 | 3.28 | 65 | 3.24 | 61 | 3.80 | 61 | 3.70 | 61 | 3.70 | 60 | 3.71 | 56 | 3.50 | 61 | 3.75 |
| P, Inorganic ion transport and metabolism | 85 | 4.92 | 81 | 4.03 | 88 | 4.20 | 83 | 4.13 | 84 | 4.18 | 72 | 4.49 | 75 | 4.55 | 75 | 4.55 | 75 | 4.64 | 59 | 3.69 | 74 | 4.55 |
| Q, Secondary metabolites biosynthesis, transport and catabolism | 18 | 1.04 | 16 | 0.80 | 18 | 0.86 | 18 | 0.90 | 18 | 0.90 | 12 | 0.75 | 12 | 0.73 | 12 | 0.73 | 12 | 0.74 | 11 | 0.69 | 12 | 0.74 |
| T, Signal transduction mechanisms | 35 | 2.03 | 34 | 1.69 | 36 | 1.72 | 34 | 1.69 | 35 | 1.74 | 30 | 1.87 | 30 | 1.82 | 30 | 1.82 | 30 | 1.86 | 27 | 1.69 | 30 | 1.85 |
| U, Intracellular trafficking, secretion, and vesicular transport | 22 | 1.27 | 21 | 1.05 | 23 | 1.10 | 20 | 1.00 | 20 | 1.00 | 20 | 1.25 | 20 | 1.21 | 21 | 1.27 | 21 | 1.30 | 21 | 1.31 | 21 | 1.29 |
| V, Defence mechanisms | 42 | 2.43 | 38 | 1.89 | 43 | 2.05 | 37 | 1.84 | 36 | 1.79 | 17 | 1.06 | 24 | 1.45 | 19 | 1.15 | 16 | 0.99 | 19 | 1.19 | 19 | 1.17 |
| S, Function unknown | 311 | 18.02 | 304 | 15.14 | 310 | 14.79 | 317 | 15.77 | 308 | 15.33 | 259 | 16.15 | 263 | 15.94 | 260 | 15.78 | 259 | 16.02 | 238 | 14.88 | 258 | 15.88 |
| COG unknown | 391 | 4.46 | 380 | 18.92 | 421 | 20.09 | 364 | 18.11 | 384 | 19.11 | 222 | 13.84 | 235 | 14.24 | 244 | 14.81 | 234 | 14.47 | 367 | 22.94 | 237 | 14.58 |

**Supplementary Table S8.** CRISPR arrays identified by CRISPRDetect v2.4 in analysed Antarctic *Rothia* strains.

| **Strain** | **Contig** | **Start** | **End** | **DR consensus** | **DR length** | **No. of repeats** |
| --- | --- | --- | --- | --- | --- | --- |
| ***R. ornithocola* sp. nov.** |  | | | | | |
| P4278 | 7 | 136878 | 139041 | CTCTTCCCCGCGTAGGCGGGGGTATTTCC | 29 | 36 |
| P5758 = CCM 9416 | 1 | 13742 | 14605 | CAAGTCTATCTGGGTTCAGAGGTGATAGTTCCCAAC | 36 | 14 |
| P5764 | - | - | - | - | - | - |
| P5766 | 31 | 60470 | 62742 | CAAGTCTATCTGGGTTCAGAGGTGATAGTTCCCAAC | 36 | 36 |
| P5771^T^ = CCM 9417^T^ | 1 | 21265 | 21535 | CTCTTCCCCGCGTAGGCGGGGGTATTTCC | 29 | 5 |
| ***R. pygoscelis* sp. nov.** |  | | | | | |
| P6271 | 1 | 1711 | 5830 | CTCTTCCCCGTACACACGGGGGTATTTCC | 29 | 68 |
| P7182^T^ = CCM 9418^T^ | 1 | 224951 | 223457 | CTCTTCCCCACGCCAGTGGGGGTAATTCC | 29 | 25 |
| P7208 | 1 | 55040 | 51414 | CTCTTCTCCGTACGCACGGAGGTATTTCC | 29 | 60 |
| ***R. antarctica* sp. nov.** |  | | | | | |
| P7162^T^ = CCM 9419^T^ | 1 | 39368 | 37993 | CTCTTCCCCACGCCAGTGGGGGTAATTCC | 29 | 23 |
| P7181 | 1 | 65413 | 65248 | CCAGCCTCAAGAACCACCAACACC | 24 | 3 |
|  | 1 | 104591 | 109925 | CTCTTCCCCGTACACACGGGGGTATTTCC | 29 | 88 |
| P13129 | 1 | 23641 | 18418 | CTCTTCCCCGTACACACGGGGGTATTTCC | 29 | 86 |

**Supplementary Table S9.** Restriction-modification (R-M) systems in analysed representative strains. Genes are listed in order R (restriction endonuclease), M (DNA methyltransferase), and S (specificity subunit).

| **Strain** | **Type** | **Gene** |
| --- | --- | --- |
| ***Rothia ornithocola* sp. nov.** |  | |
| P4278 | I | pgaptmp_000483, pgaptmp_000485, pgaptmp_000486 |
|  | I | pgaptmp_000781, pgaptmp_000777, pgaptmp_000778 |
|  | IV | pgaptmp_000657 |
|  | IV | pgaptmp_000221 |
| P5758 (= CCM 9416) | I | pgaptmp_001881, -, pgaptmp_001882 |
|  | II | pgaptmp_001076, pgaptmp_001075 |
|  | IIG | pgaptmp_001409 |
| P5764 | I | pgaptmp_001441, pgaptmp_001444, pgaptmp_001442 |
|  | I | pgaptmp_001906, pgaptmp_001902, pgaptmp_001903 |
|  | IIG | pgaptmp_001266 |
| P5766 | I | -, pgaptmp_000876, pgaptmp_000875 |
|  | II | pgaptmp_000453, pgaptmp_000454 |
|  | II | pgaptmp_000632, pgaptmp_000633 |
|  | IIG | pgaptmp_001662 |
| P5771^T^ (= CCM 9417^T^) | I | pgaptmp_001484, pgaptmp_001488, pgaptmp_001487 |
|  | II | pgaptmp_000127, pgaptmp_000126 |
|  | IV | pgaptmp_000950 |
| ***Rothia pygoscelis* sp. nov.** |  | |
| P6271 | II | pgaptmp_000050, pgaptmp_000051 |
| P7182^T^ (= CCM 9418^T^) | - | - |
| P7208 | II | pgaptmp_000993, pgaptmp_000992 |
|  | IIG | pgaptmp_000109 |
| ***Rothia antarctica* sp. nov.** |  | |
| P7162^T^ (= CCM 9419^T^) | II | pgaptmp_000770, pgaptmp_000769 |
| P7181 | I | pgaptmp_001636, pgaptmp_001634, pgaptmp_001635 |
|  | II | pgaptmp_001481, pgaptmp_001480 |
|  | II | pgaptmp_001650, pgaptmp_001649 |
|  | III | pgaptmp_000696, pgaptmp_000697 |
| P13129 | II | pgaptmp_000775, pgaptmp_000776 |
|  | II | pgaptmp_000425, pgaptmp_000426 |
|  | III | pgaptmp_001002, pgaptmp_001003 |

**Supplementary Table S10.** Summary of BGCs identified by antiSMASH 8.0.0 in the Antarctic bird-associated *Rothia* strains, including cluster types, predicted products, similarity confidence and completeness.

| **Strain ID** | **Region** | **BGC Type** | **Putative compound (MIBiG)** | **Similarity confidence**  **(MIBiG)** | **Edge**  **position** | **Incomplete**  **BGCs^a^** |
| --- | --- | --- | --- | --- | --- | --- |
| ***R. ornithocola* sp. nov.** | | | | | | |
| P5758 = CCM 9416 | 1.1 | RiPP-like |  |  | N | N |
|  | 1.2 | RRE-containing cluster |  |  | N | N |
|  | 2.1 | Terpene precursor, betalactone |  |  | N | N |
|  | 11.1 | Terpene precursor |  |  | N | N |
|  | 12.1 | NRP-metallophore, NRPS | enterobactin | medium | N | N |
| P5771^T^ = CCM 9417^T^ | 1.1 | Terpene precursor |  |  |  |  |
|  | 1.2 | NRP-metallophore, NRPS | enterobactin | medium | N | N |
|  | 10.1 | RRE-containing cluster |  |  | N | N |
|  | 11.1 | Terpene precursor, betalactone |  |  | N | N |
| P5766 | 12.1 | Terpene precursor, betalactone |  |  |  |  |
|  | 23.1 | RRE-containing cluster |  |  |  |  |
|  | 30.1 | Terpene precursor |  |  | Y | N |
|  | 30.2 | NRP-metallophore, NRPS | enterobactin | medium | Y | Y |
| P4278 | 1.1 | Terpene precursor, betalactone |  |  | N | N |
|  | 3.1 | NRP-metallophore, NRPS | enterobactin | medium | N | N |
|  | 3.2 | Terpene precursor |  |  | N | N |
|  | 5.1 | RRE-containing cluster |  |  | N | N |
| P5764 | 1.1 | NRP-metallophore, NRPS | enterobactin | medium | N | N |
|  | 12.1 | RRE-containing cluster | kosinostatin |  | N | N |
|  | 14.1 | betalactone | - | - | N | N |
| ***R. pygoscelis* sp. nov.** | | | | | | |
| P7182^T^ = CCM 9418^T^ | 1.1 | NRPS | enterobactin | high | N | N |
|  | 3.1 | Terpene precursor, betalactone |  |  | N | N |
|  | 4.1 | RRE-containing cluster |  |  | N | N |
| P6271 | 1.1 | NRPS | enterobactin | high | N | N |
|  | 10.1 | Terpene precursor, betalactone |  |  | N | N |
|  | 16.1 | RRE-containing cluster |  |  | Y | N |
| P7208 | 1.1 | NRPS | enterobactin | medium | N | N |
|  | 11.1 | Terpene precursor, betalactone |  |  | N | N |
| ***R. antarctica* sp. nov.** | | | | | | |
| P7162^T^ = CCM 9419^T^ | 5.1 | Terpene precursor, betalactone |  |  |  |  |
|  | 7.1 | NRPS | enterobactin | high | N | N |
|  | 10.1 | RRE-containing cluster |  |  | N | N |
| P7181 | 12.1 | NRPS | enterobactin | high | N | N |
|  | 19.1 | Terpene precursor, betalactone |  |  | N | N |
|  | 22.1 | RRE-containing cluster |  |  | Y | N |
| P13129 | 7.1 | Betalactone |  |  | N | N |
|  | 9.1 | NRPS | enterobactin | high | N | N |
|  | 11.1 | RRE-containing cluster |  |  | N | N |

RiPP, ribosomally synthetised and posttranslationally modified peptide; RRE, restriction recognition element; NRPS, Non-ribosomal peptide synthetase; NRP-metallophore, Non-ribosomal peptide metallophore

^a^N, no; Y, yes

**Supplementary Table S11.** Results of the Branchwater search of all 11 Antarctic bird-associated *Rothia* genomes against publicly available metagenomes in the NCBI SRA. Only hits with k-mer-based containment ≥ 0.10 are reported.

| **Strain** | **Accession** | **containment** | **cANI** | **Sample collection date** | **Country** | **Sample** | **Coordinates** |
| --- | --- | --- | --- | --- | --- | --- | --- |
| ***Rothia ornithocola* sp. nov.** | | |  |  |  |  |  |
| **P5764** | SRR16604660 | 0.29 | 0.94 | 22.10.2015 | Singapore | air metagenome | [1.320805,103.706081] |
|  | SRR21898915 | 0.27 | 0.94 | 01.03.2021 | China | metagenome | [34.79542,113.691061] |
|  | SRR26472078 | 0.23 | 0.93 | 01.01.2021 | China | wastewater metagenome | [29.54,121.37] |
|  | SRR19577158 | 0.22 | 0.93 | 10.06.2019 | China | gut metagenome |  |
|  | SRR27143223 | 0.22 | 0.93 | 14.05.2022 | China | gut metagenome | [32.24,119.35] |
|  | SRR19576871 | 0.21 | 0.93 | 10.06.2019 | China | gut metagenome |  |
|  | SRR26472077 | 0.2 | 0.93 | 01.01.2021 | China | wastewater metagenome | [29.54,121.38] |
|  | SRR19577223 | 0.19 | 0.92 | 10.06.2019 | China | gut metagenome |  |
|  | SRR20702436 | 0.17 | 0.92 | 05.09.2021 | North Korea | wastewater metagenome | [40.096,124.377] |
|  | SRR26472079 | 0.15 | 0.91 | 01.01.2021 | China | feces metagenome | [29.54,121.36] |
|  | SRR12918224 | 0.14 | 0.91 | 13.10.2017 | USA | soil metagenome | [41.896,-89.3636] |
|  | SRR12349262 | 0.14 | 0.91 | 12.07.2017 | China | gut metagenome |  |
|  | SRR21527946 | 0.12 | 0.9 | 22.06.2018 | USA | metagenome | [41.35,-72.41] |
|  | SRR26472080 | 0.11 | 0.9 | 01.01.2021 | China | feces metagenome | [29.54,121.35] |
|  | SRR15170210 | 0.11 | 0.9 | 28.05.2019 | USA | soil metagenome | [40.6529,-104.9995] |
|  | SRR12349273 | 0.1 | 0.9 | 12.07.2017 | China | gut metagenome |  |
|  | SRR12349274 | 0.1 | 0.9 | 12.07.2017 | China | gut metagenome |  |
|  | SRR27180767 | 0.1 | 0.9 | 01.11.2020 | China | feces metagenome | [24.9,102.6] |
|  | SRR27143228 | 0.1 | 0.89 | 12.07.2022 | China | gut metagenome | [32.24,119.3] |
|  | SRR13697452 | 0.1 | 0.89 | 31.05.2019 | USA | biocrust metagenome |  |
|  | SRR21020893 | 0.1 | 0.89 | 19.02.2018 | United Kingdom | soil metagenome | [57.18,-2.21] |
| **CCM 9416** | SRR16604660 | 0.3 | 0.94 | 22.10.2015 | Singapore | air metagenome | [1.320805,103.706081] |
|  | SRR21898915 | 0.28 | 0.94 | 01.03.2021 | China | metagenome | [34.79542,113.691061] |
|  | SRR26472078 | 0.23 | 0.93 | 01.01.2021 | China | wastewater metagenome | [29.54,121.37] |
|  | SRR27143223 | 0.22 | 0.93 | 14.05.2022 | China | gut metagenome | [32.24,119.35] |
|  | SRR19577158 | 0.21 | 0.93 | 10.06.2019 | China | gut metagenome |  |
|  | SRR26472077 | 0.21 | 0.93 | 01.01.2021 | China | wastewater metagenome | [29.54,121.38] |
|  | SRR19576871 | 0.21 | 0.93 | 10.06.2019 | China | gut metagenome |  |
|  | SRR19577223 | 0.19 | 0.92 | 10.06.2019 | China | gut metagenome |  |
|  | SRR20702436 | 0.17 | 0.92 | 05.09.2021 | North Korea | wastewater metagenome | [40.096,124.377] |
|  | SRR26472079 | 0.15 | 0.91 | 01.01.2021 | China | feces metagenome | [29.54,121.36] |
|  | SRR12349262 | 0.14 | 0.91 | 12.07.2017 | China | gut metagenome |  |
|  | SRR12918224 | 0.14 | 0.91 | 13.10.2017 | USA | soil metagenome | [41.896,-89.3636] |
|  | SRR21527946 | 0.12 | 0.9 | 22.06.2018 | USA | metagenome | [41.35,-72.41] |
|  | SRR15170210 | 0.11 | 0.9 | 28.05.2019 | USA | soil metagenome | [40.6529,-104.9995] |
|  | SRR26472080 | 0.11 | 0.9 | 01.01.2021 | China | feces metagenome | [29.54,121.35] |
|  | SRR15532679 | 0.1 | 0.9 | 30.10.2013 | USA | wetland metagenome | [41.3778,-82.5108] |
|  | SRR12349274 | 0.1 | 0.9 | 12.07.2017 | China | gut metagenome |  |
|  | SRR15532681 | 0.1 | 0.9 | 30.10.2013 | USA | wetland metagenome | [41.384,-82.513] |
|  | SRR13697452 | 0.1 | 0.9 | 31.05.2019 | USA | biocrust metagenome |  |
|  | SRR16604629 | 0.1 | 0.9 | 29.10.2015 | Singapore | air metagenome | [1.320805,103.706081] |
|  | SRR15532680 | 0.1 | 0.9 | 30.10.2013 | USA | wetland metagenome | [41.3778,-82.5108] |
|  | SRR27143228 | 0.1 | 0.9 | 12.07.2022 | China | gut metagenome | [32.24,119.3] |
|  | SRR12349273 | 0.1 | 0.9 | 12.07.2017 | China | gut metagenome |  |
|  | SRR27180767 | 0.1 | 0.9 | 01.11.2020 | China | feces metagenome | [24.9,102.6] |
|  | SRR408201 | 0.1 | 0.89 |  | NP | soil metagenome |  |
|  | SRR490139 | 0.1 | 0.89 |  | NP | soil metagenome |  |
|  | SRR490148 | 0.1 | 0.89 |  | NP | soil metagenome |  |
|  | SRR490140 | 0.1 | 0.89 |  | NP | soil metagenome |  |
| **P5766** | SRR16604660 | 0.3 | 0.94 | 22.10.2015 | Singapore | air metagenome | [1.320805,103.706081] |
|  | SRR21898915 | 0.28 | 0.94 | 01.03.2021 | China | metagenome | [34.79542,113.691061] |
|  | SRR26472078 | 0.23 | 0.93 | 01.01.2021 | China | wastewater metagenome | [29.54,121.37] |
|  | SRR19577158 | 0.22 | 0.93 | 10.06.2019 | China | gut metagenome |  |
|  | SRR19576871 | 0.22 | 0.93 | 10.06.2019 | China | gut metagenome |  |
|  | SRR27143223 | 0.22 | 0.93 | 14.05.2022 | China | gut metagenome | [32.24,119.35] |
|  | SRR26472077 | 0.21 | 0.93 | 01.01.2021 | China | wastewater metagenome | [29.54,121.38] |
|  | SRR19577223 | 0.2 | 0.93 | 10.06.2019 | China | gut metagenome |  |
|  | SRR20702436 | 0.17 | 0.92 | 05.09.2021 | North Korea | wastewater metagenome | [40.096,124.377] |
|  | SRR26472079 | 0.16 | 0.92 | 01.01.2021 | China | feces metagenome | [29.54,121.36] |
|  | SRR12349262 | 0.15 | 0.91 | 12.07.2017 | China | gut metagenome |  |
|  | SRR12918224 | 0.14 | 0.91 | 13.10.2017 | USA | soil metagenome | [41.896,-89.3636] |
|  | SRR21527946 | 0.12 | 0.9 | 22.06.2018 | USA | metagenome | [41.35,-72.41] |
|  | SRR12349273 | 0.11 | 0.9 | 12.07.2017 | China | gut metagenome |  |
|  | SRR26472080 | 0.11 | 0.9 | 01.01.2021 | China | feces metagenome | [29.54,121.35] |
|  | SRR15170210 | 0.11 | 0.9 | 28.05.2019 | USA | soil metagenome | [40.6529,-104.9995] |
|  | SRR408201 | 0.1 | 0.9 |  | NP | soil metagenome |  |
|  | SRR27180767 | 0.1 | 0.9 | 01.11.2020 | China | feces metagenome | [24.9,102.6] |
|  | SRR27143228 | 0.1 | 0.9 | 12.07.2022 | China | gut metagenome | [32.24,119.3] |
|  | SRR15532681 | 0.1 | 0.9 | 30.10.2013 | USA | wetland metagenome | [41.384,-82.513] |
|  | SRR13697452 | 0.1 | 0.9 | 31.05.2019 | USA | biocrust metagenome |  |
|  | SRR15532679 | 0.1 | 0.9 | 30.10.2013 | USA | wetland metagenome | [41.3778,-82.5108] |
|  | SRR12349274 | 0.1 | 0.9 | 12.07.2017 | China | gut metagenome |  |
|  | SRR15532680 | 0.1 | 0.9 | 30.10.2013 | USA | wetland metagenome | [41.3778,-82.5108] |
|  | SRR490139 | 0.1 | 0.89 |  | NP | soil metagenome |  |
|  | SRR490140 | 0.1 | 0.89 |  | NP | soil metagenome |  |
|  | SRR16604629 | 0.1 | 0.89 | 29.10.2015 | Singapore | air metagenome | [1.320805,103.706081] |
|  | SRR23971535 | 0.1 | 0.89 | 12.01.2020 | China | metagenome | [39.91,116.41] |
| **CCM 9417** | SRR16604660 | 0.32 | 0.95 | 22.10.2015 | Singapore | air metagenome | [1.320805,103.706081] |
|  | SRR21898915 | 0.27 | 0.94 | 01.03.2021 | China | metagenome | [34.79542,113.691061] |
|  | SRR26472078 | 0.23 | 0.93 | 01.01.2021 | China | wastewater metagenome | [29.54,121.37] |
|  | SRR19577158 | 0.22 | 0.93 | 10.06.2019 | China | gut metagenome |  |
|  | SRR26472077 | 0.22 | 0.93 | 01.01.2021 | China | wastewater metagenome | [29.54,121.38] |
|  | SRR27143223 | 0.22 | 0.93 | 14.05.2022 | China | gut metagenome | [32.24,119.35] |
|  | SRR19576871 | 0.21 | 0.93 | 10.06.2019 | China | gut metagenome |  |
|  | SRR19577223 | 0.19 | 0.92 | 10.06.2019 | China | gut metagenome |  |
|  | SRR20702436 | 0.17 | 0.92 | 05.09.2021 | North Korea | wastewater metagenome | [40.096,124.377] |
|  | SRR26472079 | 0.15 | 0.91 | 01.01.2021 | China | feces metagenome | [29.54,121.36] |
|  | SRR12349262 | 0.14 | 0.91 | 12.07.2017 | China | gut metagenome |  |
|  | SRR12918224 | 0.14 | 0.91 | 13.10.2017 | USA | soil metagenome | [41.896,-89.3636] |
|  | SRR21527946 | 0.12 | 0.9 | 22.06.2018 | USA | metagenome | [41.35,-72.41] |
|  | SRR15170210 | 0.11 | 0.9 | 28.05.2019 | USA | soil metagenome | [40.6529,-104.9995] |
|  | SRR26472080 | 0.11 | 0.9 | 01.01.2021 | China | feces metagenome | [29.54,121.35] |
|  | SRR490139 | 0.1 | 0.9 |  | NP | soil metagenome |  |
|  | SRR15532679 | 0.1 | 0.9 | 30.10.2013 | USA | wetland metagenome | [41.3778,-82.5108] |
|  | SRR12349274 | 0.1 | 0.9 | 12.07.2017 | China | gut metagenome |  |
|  | SRR13697452 | 0.1 | 0.9 | 31.05.2019 | USA | biocrust metagenome |  |
|  | SRR16604629 | 0.1 | 0.9 | 29.10.2015 | Singapore | air metagenome | [1.320805,103.706081] |
|  | SRR27143228 | 0.1 | 0.9 | 12.07.2022 | China | gut metagenome | [32.24,119.3] |
|  | SRR12349273 | 0.1 | 0.9 | 12.07.2017 | China | gut metagenome |  |
|  | SRR27180767 | 0.1 | 0.9 | 01.11.2020 | China | feces metagenome | [24.9,102.6] |
|  | SRR15532680 | 0.1 | 0.89 | 30.10.2013 | USA | wetland metagenome | [41.3778,-82.5108] |
|  | SRR490130 | 0.1 | 0.89 |  | NP | soil metagenome |  |
|  | SRR490140 | 0.1 | 0.89 |  | NP | soil metagenome |  |
|  | SRR408201 | 0.1 | 0.89 |  | NP | soil metagenome |  |
| **P4278** | SRR16604660 | 0.31 | 0.95 | 22.10.2015 | Singapore | air metagenome | [1.320805,103.706081] |
|  | SRR21898915 | 0.28 | 0.94 | 01.03.2021 | China | metagenome | [34.79542,113.691061] |
|  | SRR26472078 | 0.24 | 0.93 | 01.01.2021 | China | wastewater metagenome | [29.54,121.37] |
|  | SRR27143223 | 0.22 | 0.93 | 14.05.2022 | China | gut metagenome | [32.24,119.35] |
|  | SRR19577158 | 0.22 | 0.93 | 10.06.2019 | China | gut metagenome |  |
|  | SRR26472077 | 0.22 | 0.93 | 01.01.2021 | China | wastewater metagenome | [29.54,121.38] |
|  | SRR19576871 | 0.21 | 0.93 | 10.06.2019 | China | gut metagenome |  |
|  | SRR19577223 | 0.2 | 0.93 | 10.06.2019 | China | gut metagenome |  |
|  | SRR20702436 | 0.17 | 0.92 | 05.09.2021 | North Korea | wastewater metagenome | [40.096,124.377] |
|  | SRR26472079 | 0.15 | 0.91 | 01.01.2021 | China | feces metagenome | [29.54,121.36] |
|  | SRR12349262 | 0.14 | 0.91 | 12.07.2017 | China | gut metagenome |  |
|  | SRR12918224 | 0.14 | 0.91 | 13.10.2017 | USA | soil metagenome | [41.896,-89.3636] |
|  | SRR21527946 | 0.12 | 0.91 | 22.06.2018 | USA | metagenome | [41.35,-72.41] |
|  | SRR15170210 | 0.11 | 0.9 | 28.05.2019 | USA | soil metagenome | [40.6529,-104.9995] |
|  | SRR26472080 | 0.11 | 0.9 | 01.01.2021 | China | feces metagenome | [29.54,121.35] |
|  | SRR12349274 | 0.1 | 0.9 | 12.07.2017 | China | gut metagenome |  |
|  | SRR13697452 | 0.1 | 0.9 | 31.05.2019 | USA | biocrust metagenome |  |
|  | SRR16604629 | 0.1 | 0.9 | 29.10.2015 | Singapore | air metagenome | [1.320805,103.706081] |
|  | SRR27143228 | 0.1 | 0.9 | 12.07.2022 | China | gut metagenome | [32.24,119.3] |
|  | SRR12349273 | 0.1 | 0.9 | 12.07.2017 | China | gut metagenome |  |
|  | SRR27180767 | 0.1 | 0.9 | 01.11.2020 | China | feces metagenome | [24.9,102.6] |
|  | SRR15532679 | 0.1 | 0.89 | 30.10.2013 | USA | wetland metagenome | [41.3778,-82.5108] |
|  | SRR15532680 | 0.1 | 0.89 | 30.10.2013 | USA | wetland metagenome | [41.3778,-82.5108] |
| ***Rothia pygoscelis* sp. nov.** | | |  |  |  |  |  |
| **P6271** | SRR13514086 | 0.11 | 0.9 |  | USA | lichen metagenome |  |
|  | SRR16526563 | 0.1 | 0.89 | 11.10.2019 | USA | bioreactor sludge metagenome | [43.0738,-89.4139] |
| **P7208** | SRR13514086 | 0.11 | 0.9 |  | USA | lichen metagenome |  |
| **CCM 9418** | SRR13514086 | 0.11 | 0.9 |  | USA | lichen metagenome |  |
| ***Rothia antarctica* sp. nov.** | | |  |  |  |  |  |
| **CCM 9419** | SRR13514086 | 0.11 | 0.9 |  | USA | lichen metagenome |  |
|  | SRR16526563 | 0.1 | 0.9 | 11.10.2019 | USA | bioreactor sludge metagenome | [43.0738,-89.4139] |
| **P7181** | SRR13514086 | 0.11 | 0.9 |  | USA | lichen metagenome |  |
|  | SRR16526563 | 0.1 | 0.9 | 11.10.2019 | USA | bioreactor sludge metagenome | [43.0738,-89.4139] |
| **P13129** | SRR13514086 | 0.11 | 0.9 |  | USA | lichen metagenome |  |

NP, not provided.
